# The family Conopidae (Diptera) in Egypt and Saudi Arabia

**DOI:** 10.3897/BDJ.9.e60287

**Published:** 2021-01-13

**Authors:** Magdi El-Hawagry, Ahmed Mostafa Soliman, Hathal Mohammed Al Dhafer

**Affiliations:** 1 Entomology Department, Faculty of Science, Cairo University, Giza, Egypt Entomology Department, Faculty of Science, Cairo University Giza Egypt; 2 Plant Protection Department, College of Food and Agriculture Sciences, King Saud University, PO BOX 2460, Riyadh, Saudi Arabia Plant Protection Department, College of Food and Agriculture Sciences, King Saud University, PO BOX 2460 Riyadh Saudi Arabia; 3 Al-Azhar University, Faculty of Science, Cairo, Egypt Al-Azhar University, Faculty of Science Cairo Egypt

**Keywords:** thick-headed flies, local distribution, dates of collection, new records

## Abstract

**Background:**

The present study is one in a series of planned studies aiming to catalogue the whole order Diptera in both Egypt and Saudi Arabia.

**New information:**

All known Egyptian and Saudi Arabian conopid taxa are systematically catalogued in the present study. Three species are recorded herein for the first time from Saudi Arabia: Conops (Asiconops) elegans Meigen, 1804 and *Thecophora atra* (Fabricius, 1775) (Al-Baha region, south-western of Saudi Arabia), and Conops (Conops) quadrifasciatus De Geer, 1776 (Tabuk region, north-western of Saudi Arabia). *Physocephala variegata* (Meigen, 1924) is also recorded for the first time from Gebel Elba, the south-eastern triangle of Egypt. Considering that Gebel Elba in Egypt and Al-Baha in Saudi Arabia are affiliated to the Afrotropical Region, this is the first time *Physocephala variegata* and *Thecophora atra* have been recorded from the Afrotropical Region. An updated taxonomy, world and local distributions, dates of collection and some coloured photographs are provided.

## Introduction

The Conopidae, commonly known as thick-headed flies, are an interesting brachycerous family of usually large-sized wasp-like flies. Many conopids are most frequently found at flowers feeding on nectar using their long proboscis and considered as important pollinators. The majority of conopid larvae are obligatory endoparasitoids of adult Hymenoptera, such as bees and wasps. Adult conopid females aggressively intercept their hymenopterous hosts in the field to deposit their eggs (Freeman (1966), Gibson and Skevington (2013), Pape et al. (2011), Stuke (2017), Azmy et al. (2016)). The family Conopidae is represented by 808 valid species assigned to 57 genera worldwide (Stuke 2017). Conopids are found in all parts of the world, except Antarctica and the Pacific Islands (Gibson et al. 2013).

**Figure 1.**
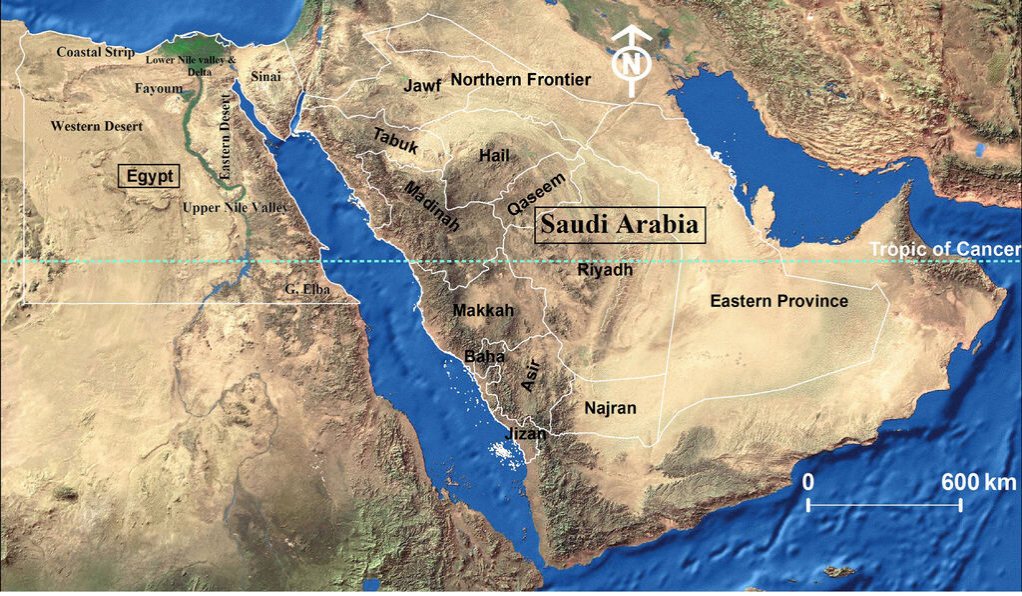
A satellite map of Egypt and Saudi Arabia.

Egypt and Saudi Arabia are two neighbouring Middle Eastern countries, separated by the Gulf of Aqaba and the Red Sea (Fig. 1). Both countries are located at the junction of the Palaearctic and the Afrotropical Regions and the faunal affiliation of them is mainly Palaearctic, except Gebel Elba, the south-eastern triangle of Egypt and the south-western part of Saudi Arabia, south to the Tropic of Cancer, which are considered as Afrotropical (Hölzel 1998, Wallace 1876, El-Hawagry and Gilbert 2014, El-Hawagry et al. 2020).

Some previous studies have been carried out in Egypt on the taxonomy and faunistics of the family Conopidae, including Kröber (1924), Kröber (1925), Kröber (1927), Kröber (1929), Steyskal and El-Bialy (1967), Mohammed and Negm (1991), Azmy (2016) and Azmy et al. (2016), in addition to some miscellaneous studies which described some new species from Egypt as Kröber (1915a), Wiedemann (1830), Macquart (1835) and Rondani (1850). On the other hand, no previous faunistic or taxonomic studies on the family Conopidae have been carried out before in Saudi Arabia and only a few species have been described or recorded there amid some miscellaneous studies, such as Macquart (1851), Kröber (1915a), Zimina (1963), Walker and Pittaway (1987), Abu-Zoherah et al. (1993), Stuke and Clements (2008), El-Hawagry et al. (2016) and El-Hawagry et al. (2017).

The abundance and diversity of conopid species in both Egypt and Saudi Arabia seem to be low (17 species in Egypt and 12 species in Saudi Arabia) comparable with the total number of species known from the Palaearctic Region (173 species) and from the Afrotropical Region (187 species) (Stuke 2017). However, this should be taken with caution, since the family Conopidae seems to lack sampling efforts, especially in Saudi Arabia. This is most likely due to the fact that documentation of biological diversity in Saudi Arabia began in the second half of the 1960s and no systematic studies on the Conopidae have been previously conducted in this country. We think that the faunistic data of these flies in Egypt and Saudi Arabia is still scanty and more efforts would be highly required.

The taxonomy of Conopidae in both Egypt and Saudi Arabia is updated in the present study. Twenty species assigned to five genera and three subfamilies (Conopinae, Myopinae and Zodioninae) are treated (Table 1). Three species, Conops (Asiconops) elegans Meigen, 1804, Conops (Conops) quadrifasciatus De Geer, 1776 and *Thecophora atra* (Fabricius, 1775), are recorded for the first time from Saudi Arabia.

*Physocephala variegata* (Meigen) is recorded for the first time from Gebel Elba, the south-eastern triangle of Egypt and *Thecophora atra* (Fabricius) is recorded for the first time from the south-western part of Saudi Arabia. Considering that Gebel Elba in Egypt and the south-western part in Saudi Arabia including Al-Baha Region are affiliated to the Afrotropical Region, this is the first time that these two species from the Afrotropical Region have been recorded.

Walker (1871) described *Conops auratus* from “Hor Tamanib” which belonged to Egypt at the time of description; however, it lies now in Sudan. Steyskal and El-Bialy (1967) inaccurately listed this species as an Egyptian species, based mainly on the misleading type locality. Thus, this species is excluded from the Egyptian list in the present study.

This study is one in a series of taxonomic studies on different Egyptian and Saudi Arabian dipteran taxa aiming to catalogue the whole order Diptera in the two countries.

## Materials and methods

**Data sources**. Data of the present study are obtained from three main sources: 1. Specimens preserved in the Egyptian and Saudi Arabian insect collections and museums, namely: Efflatoun Bey’s collection, Cairo University, Egypt (EFC); Collection of Entomological Society of Egypt (ESEC); King Saud University Museum of Arthropods, Saudi Arabia (KSMA) and Personal collection M. El-Hawagry (MSHC). 2. Material collected by the authors and their co-workers. 3. Previous studies on the Egyptian and Saudi Arabian Conopidae. The majority of specimens preserved in EFC and ESEC were determined by Dr. Otto Kröber. However, all identifications have been rechecked by the first author using Kröber (1927), Stuke and Clements (2008), Azmy (2016) and Azmy et al. (2016). Photos of *Thecophora atra* have been checked by Dr. Jens-Hermann Stuke (personal communication).

**Study area**. Ecologists divide Egypt into eight ecological zones: the Coastal Strip (= Mediterranean Coastal Strip), Lower Nile Valley and Delta, Upper Nile Valley, Fayoum, Eastern Desert, Western Desert, Sinai and Gebel Elba (El-Hawagry and Gilbert 2014). These ecological zones are used in the present study in the sections of local distribution. However, Saudi Arabia is not divided into ecological zones by ecologists, so the administrative divisions (also called regions, provinces or emirates) are used instead, namely: Makkah, Riyadh, Eastern Province, Asir, Jazan, Al-Madinah, Al-Qaseem, Tabuk, Hail, Najran, Al-Jawf, Al-Baha and Northern Frontier (Fig. 1) (El-Hawagry et al. 2020).

**Classification.** The classification and nomenclature in the present study basically follows that used in Stuke (2017), in addition to the tribal arrangement suggested by Gibson and Skevington (2013). Full lists of synonyms for genera and species are not given in the present study as they are already listed in world and regional catalogues; however, we listed only the synonyms of species that have been mentioned in previous Egyptian and Saudi Arabian literature.

**World distribution**. Stuke (2017) has collected all sources of world distribution of species and it is used here as our basic source. The additional sources, if any, are given between parentheses after each record.

**Local distribution and dates of collection**. Localities within each Egyptian ecological zone or each Saudi Arabian administrative region are arranged alphabetically and written after a colon following each zone or region, followed by the dates of collection between parentheses. Sources for the local distribution of each species in each country are given between square brackets at the end of the section. Coordinates of Egyptian and Saudi Arabian localities of the family Conopidae are listed (Table 2). Local distribution maps of species based on all available records are given, using SimpleMappr (Shorthouse 2010).

Abbreviations used:

- **AF**, Afrotropical.
- **EFC**, Efflatoun Bey’s collection, Department of Entomology, Faculty of Science, Cairo University, Egypt.
- **ESEC**, Collection of Entomological Society of Egypt, Cairo, Egypt.
- **KSMA**, King Saud University Museum of Arthropods, Riyadh, Saudi Arabia.
- **MSHC**, Personal collection M. El-Hawagry.
- **OR**, Oriental.
- **PA**, Palearctic.

## Checklists

### The catalogue

#### CONOPIDAE

2886EBB0-F4EB-58D7-8BF6-EAD3A69B6FFE

#### CONOPINAE

A9C02511-9D07-57D5-BD4F-EA81E057285E

#### CONOPINI

07DBF252-FE1F-5C41-96FD-2D0273F8B1BC

#### Conops

Linnaeus

BC310CE2-5316-5909-895B-815792C7DEDB

https://www.gbif.org/species/1444664

Conops ***Conops*** Linnaeus, 1758: 604. Type species: *Conops flavipes* Linnaeus, by subsequent designation of Curtis (1831).

#### Asiconops

Chen

5168C48E-6A17-5C60-AC1A-7862169A67D9

Asiconops ***Asiconops*** Chen, 1939: 171. Type species: *Conops aureomaculatus* Kröber, by original designation.

#### Conops (Asiconops) elegans

Meigen, 1804

EA1D392D-75C8-5867-AD62-3DD8AD852EAA

https://www.gbif.org/species/5076777

Conops elegans Meigen, 1804: 275. Type locality: France (Marseille).
Conops elegans var. fuscipennis Macquart 1849: 472. Type locality: Algeria.
Conops algirus Macquart 1849: 473. Type locality: Algeria (environs du Cercle de Lacalle).
Conopaejus aegyptiacus Rondani, 1850: 167. Type locality: Egypt.
Conops fuscanipennis Bigot, 1887: 32. Type locality: Italy (Sicily).
Conops elegans var. minutus Kröber, 1916: 48. Type locality: Not given (Cyprus, Limassol in Kröber 1939).

##### Materials

- **Type status:** Other material. **Occurrence:** recordedBy: Aly; sex: 2 males, 1 female; lifeStage: adult; **Taxon:** taxonID: https://www.gbif.org/species/5076777; scientificName: Conops elegans; kingdom: Animalia; phylum: Arthropoda; class: Insecta; order: Diptera; family: Conopidae; **Location:** country: Egypt; locality: Fayid; decimalLatitude: 30.323823; decimalLongitude: 32.30079; **Identification:** identifiedBy: M. El-Hawagry; dateIdentified: May 2020; **Event:** samplingProtocol: Sweeping; eventDate: 03-16-1959; **Record Level:** institutionCode: EFC
- **Type status:** Other material. **Occurrence:** recordedBy: Farag; sex: 1 female; lifeStage: adult; **Taxon:** taxonID: https://www.gbif.org/species/5076777; scientificName: Conops elegans; kingdom: Animalia; phylum: Arthropoda; class: Insecta; order: Diptera; family: Conopidae; **Location:** country: Egypt; locality: W. Garawi; decimalLatitude: 29.7833; decimalLongitude: 31.3167; **Identification:** identifiedBy: M.El-Hawagry; dateIdentified: May 2020; **Event:** samplingProtocol: Sweeping; eventDate: 05-06-1927; **Record Level:** institutionCode: EFC
- **Type status:** Other material. **Occurrence:** recordedBy: Shafik & Str.; sex: 1 male, 1 female; lifeStage: adult; **Taxon:** taxonID: https://www.gbif.org/species/5076777; scientificName: Conops elegans; kingdom: Animalia; phylum: Arthropoda; class: Insecta; order: Diptera; family: Conopidae; **Location:** country: Egypt; locality: Girza; decimalLatitude: 29.499684; decimalLongitude: 31.07380; **Identification:** identifiedBy: M. El-Hawagry; dateIdentified: May 2020; **Event:** samplingProtocol: Sweeping; eventDate: 05-16-1952; **Record Level:** institutionCode: EFC
- **Type status:** Other material. **Occurrence:** recordedBy: Str. & Sh.M.; sex: 1 male; lifeStage: adult; **Taxon:** taxonID: https://www.gbif.org/species/5076777; scientificName: Conops elegans; kingdom: Animalia; phylum: Arthropoda; class: Insecta; order: Diptera; family: Conopidae; **Location:** country: Egypt; locality: Tameyah; decimalLatitude: 29.475479; decimalLongitude: 30.95228; **Identification:** identifiedBy: M. El-Hawagry; dateIdentified: May 2020; **Event:** samplingProtocol: Sweeping; eventDate: 07-25-1948; **Record Level:** institutionCode: EFC
- **Type status:** Other material. **Occurrence:** recordedBy: Tewfik; sex: 1 male; lifeStage: adult; **Taxon:** taxonID: https://www.gbif.org/species/5076777; scientificName: Conops elegans; kingdom: Animalia; phylum: Arthropoda; class: Insecta; order: Diptera; family: Conopidae; **Location:** country: Egypt; locality: Abu-Rawash; decimalLatitude: 30.043845; decimalLongitude: 31.0929; **Identification:** identifiedBy: M. El-Hawagry; dateIdentified: May 2020; **Event:** samplingProtocol: Sweeping; eventDate: 05-17-1925; **Record Level:** institutionCode: EFC
- **Type status:** Other material. **Occurrence:** recordedBy: R.M.; sex: 2 males; lifeStage: adult; **Taxon:** taxonID: https://www.gbif.org/species/5076777; scientificName: Conops elegans; kingdom: Animalia; phylum: Arthropoda; class: Insecta; order: Diptera; family: Conopidae; **Location:** country: Egypt; locality: Abu-Rawash; decimalLatitude: 30.043845; decimalLongitude: 31.0929; **Identification:** identifiedBy: M. El-Hawagry; dateIdentified: May 2020; **Event:** samplingProtocol: Sweeping; eventDate: 09-16-1925; **Record Level:** institutionCode: EFC
- **Type status:** Other material. **Occurrence:** recordedBy: R.M.; sex: 1 female; lifeStage: adult; **Taxon:** taxonID: https://www.gbif.org/species/5076777; scientificName: Conops elegans; kingdom: Animalia; phylum: Arthropoda; class: Insecta; order: Diptera; family: Conopidae; **Location:** country: Egypt; locality: Abu-Rawash; decimalLatitude: 30.043845; decimalLongitude: 31.0929; **Identification:** identifiedBy: M. El-Hawagry; dateIdentified: May 2020; **Event:** samplingProtocol: Sweeping; eventDate: 10-13-1926; **Record Level:** institutionCode: EFC
- **Type status:** Other material. **Occurrence:** recordedBy: Mistikawi; sex: 2 males, 1 female; lifeStage: adult; **Taxon:** taxonID: https://www.gbif.org/species/5076777; scientificName: Conops elegans; kingdom: Animalia; phylum: Arthropoda; class: Insecta; order: Diptera; family: Conopidae; **Location:** country: Egypt; locality: Edfina; decimalLatitude: 31.29252; decimalLongitude: 30.51322; **Identification:** identifiedBy: M. El-Hawagry; dateIdentified: May 2020; **Event:** samplingProtocol: Sweeping; eventDate: 06-08-1914; **Record Level:** institutionCode: EFC
- **Type status:** Other material. **Occurrence:** recordedBy: Efflatoun; sex: 1 female; lifeStage: adult; **Taxon:** taxonID: https://www.gbif.org/species/5076777; scientificName: Conops elegans; kingdom: Animalia; phylum: Arthropoda; class: Insecta; order: Diptera; family: Conopidae; **Location:** country: Egypt; locality: Ezbet-Naghl; decimalLatitude: 31.1111; decimalLongitude: 32.1625; **Identification:** identifiedBy: M. El-Hawagry; dateIdentified: May 2020; **Event:** samplingProtocol: Sweeping; eventDate: 04-13-1922; **Record Level:** institutionCode: EFC
- **Type status:** Other material. **Occurrence:** recordedBy: Tewfik; sex: 1 female; lifeStage: adult; **Taxon:** taxonID: https://www.gbif.org/species/5076777; scientificName: Conops elegans; kingdom: Animalia; phylum: Arthropoda; class: Insecta; order: Diptera; family: Conopidae; **Location:** country: Egypt; locality: Ezbet-Naghl; decimalLatitude: 32.1111; decimalLongitude: 32.1625; **Identification:** identifiedBy: M. El-Hawagry; dateIdentified: May 2020; **Event:** samplingProtocol: Sweeping; eventDate: 06-06-1926; **Record Level:** institutionCode: EFC
- **Type status:** Other material. **Occurrence:** recordedBy: Alfieri; sex: 1 female; lifeStage: adult; **Taxon:** taxonID: https://www.gbif.org/species/5076777; scientificName: Conops elegans; kingdom: Animalia; phylum: Arthropoda; class: Insecta; order: Diptera; family: Conopidae; **Location:** country: Egypt; locality: Ghezirah; decimalLatitude: 30.012106; decimalLongitude: 31.21200; **Identification:** identifiedBy: M. El-Hawagry; dateIdentified: May 2020; **Event:** samplingProtocol: Sweeping; eventDate: 05-20-1920; **Record Level:** institutionCode: EFC
- **Type status:** Other material. **Occurrence:** recordedBy: Farag; sex: 1 male; lifeStage: adult; **Taxon:** taxonID: https://www.gbif.org/species/5076777; scientificName: Conops elegans; kingdom: Animalia; phylum: Arthropoda; class: Insecta; order: Diptera; family: Conopidae; **Location:** country: Egypt; locality: Helwan; decimalLatitude: 29.8500; decimalLongitude: 31.3333; **Identification:** identifiedBy: M. El-Hawagry; dateIdentified: May 2020; **Event:** samplingProtocol: Sweeping; eventDate: 04-21-1934; **Record Level:** institutionCode: EFC
- **Type status:** Other material. **Occurrence:** recordedBy: Farag; sex: 1 male; lifeStage: adult; **Taxon:** taxonID: https://www.gbif.org/species/5076777; scientificName: Conops elegans; kingdom: Animalia; phylum: Arthropoda; class: Insecta; order: Diptera; family: Conopidae; **Location:** country: Egypt; locality: Helwan; decimalLatitude: 29.8500; decimalLongitude: 31.3333; **Identification:** identifiedBy: M. El-Hawagry; dateIdentified: May 2020; **Event:** samplingProtocol: Sweeping; eventDate: 04-28-1934; **Record Level:** institutionCode: EFC
- **Type status:** Other material. **Occurrence:** recordedBy: Farag; sex: 1 female; lifeStage: adult; **Taxon:** taxonID: https://www.gbif.org/species/5076777; scientificName: Conops elegans; kingdom: Animalia; phylum: Arthropoda; class: Insecta; order: Diptera; family: Conopidae; **Location:** country: Egypt; locality: Helwan; decimalLatitude: 29.8500; decimalLongitude: 31.3333; **Identification:** identifiedBy: M. El-Hawagry; dateIdentified: May 2020; **Event:** samplingProtocol: Sweeping; eventDate: 05-26-1934; **Record Level:** institutionCode: EFC
- **Type status:** Other material. **Occurrence:** recordedBy: R.M.; sex: 1 male; lifeStage: adult; **Taxon:** taxonID: https://www.gbif.org/species/5076777; scientificName: Conops elegans; kingdom: Animalia; phylum: Arthropoda; class: Insecta; order: Diptera; family: Conopidae; **Location:** country: Egypt; locality: Kafr Hakim; decimalLatitude: 30.0808; decimalLongitude: 31.1164; **Identification:** identifiedBy: M. El-Hawagry; dateIdentified: May 2020; **Event:** samplingProtocol: Sweeping; eventDate: 10-17-1925; **Record Level:** institutionCode: EFC
- **Type status:** Other material. **Occurrence:** recordedBy: R.M.; sex: 1 female; lifeStage: adult; **Taxon:** taxonID: https://www.gbif.org/species/5076777; scientificName: Conops elegans; kingdom: Animalia; phylum: Arthropoda; class: Insecta; order: Diptera; family: Conopidae; **Location:** country: Egypt; locality: Kafr Hakim; decimalLatitude: 30.0808; decimalLongitude: 31.1164; **Identification:** identifiedBy: M. El-Hawagry; dateIdentified: May 2020; **Event:** samplingProtocol: Sweeping; eventDate: 06-08-1927; **Record Level:** institutionCode: EFC
- **Type status:** Other material. **Occurrence:** recordedBy: L.H.G; sex: 2 males; lifeStage: adult; **Taxon:** taxonID: https://www.gbif.org/species/5076777; scientificName: Conops elegans; kingdom: Animalia; phylum: Arthropoda; class: Insecta; order: Diptera; family: Conopidae; **Location:** country: Egypt; locality: Maadi; decimalLatitude: 29.957721; decimalLongitude: 31.25054; **Identification:** identifiedBy: M. El-Hawagry; dateIdentified: May 2020; **Event:** samplingProtocol: Sweeping; eventDate: 06-20-12; **Record Level:** institutionCode: EFC
- **Type status:** Other material. **Occurrence:** recordedBy: R.M.; sex: 1 female; lifeStage: adult; **Taxon:** taxonID: https://www.gbif.org/species/5076777; scientificName: Conops elegans; kingdom: Animalia; phylum: Arthropoda; class: Insecta; order: Diptera; family: Conopidae; **Location:** country: Egypt; locality: Kerdassa; decimalLatitude: 30.0297; decimalLongitude: 31.1061; **Identification:** identifiedBy: M. El-Hawagry; dateIdentified: May 2020; **Event:** samplingProtocol: Sweeping; eventDate: 06-10-1925; **Record Level:** institutionCode: EFC
- **Type status:** Other material. **Occurrence:** recordedBy: R.M.; sex: 1 female; lifeStage: adult; **Taxon:** taxonID: https://www.gbif.org/species/5076777; scientificName: Conops elegans; kingdom: Animalia; phylum: Arthropoda; class: Insecta; order: Diptera; family: Conopidae; **Location:** country: Egypt; locality: Kerdassa; decimalLatitude: 30.0297; decimalLongitude: 31.1061; **Identification:** identifiedBy: M. El-Hawagry; dateIdentified: May 2020; **Event:** samplingProtocol: Sweeping; eventDate: 10-09-1926; **Record Level:** institutionCode: EFC
- **Type status:** Other material. **Occurrence:** recordedBy: R.M.; sex: 1 female; lifeStage: adult; **Taxon:** taxonID: https://www.gbif.org/species/5076777; scientificName: Conops elegans; kingdom: Animalia; phylum: Arthropoda; class: Insecta; order: Diptera; family: Conopidae; **Location:** country: Egypt; locality: Kerdassa; decimalLatitude: 30.0297; decimalLongitude: 31.1061; **Identification:** identifiedBy: M. El-Hawagry; dateIdentified: May 2020; **Event:** samplingProtocol: Sweeping; eventDate: 10-30-1926; **Record Level:** institutionCode: EFC
- **Type status:** Other material. **Occurrence:** recordedBy: R.M.; sex: 1 male, 1 female; lifeStage: adult; **Taxon:** taxonID: https://www.gbif.org/species/5076777; scientificName: Conops elegans; kingdom: Animalia; phylum: Arthropoda; class: Insecta; order: Diptera; family: Conopidae; **Location:** country: Egypt; locality: Kerdassa; decimalLatitude: 30.0297; decimalLongitude: 31.1061; **Identification:** identifiedBy: M. El-Hawagry; dateIdentified: May 2020; **Event:** samplingProtocol: Sweeping; eventDate: 05-07-1927; **Record Level:** institutionCode: EFC
- **Type status:** Other material. **Occurrence:** recordedBy: R.M.; sex: 1 male; lifeStage: adult; **Taxon:** taxonID: https://www.gbif.org/species/5076777; scientificName: Conops elegans; kingdom: Animalia; phylum: Arthropoda; class: Insecta; order: Diptera; family: Conopidae; **Location:** country: Egypt; locality: Mansouriah; decimalLatitude: 30.1236; decimalLongitude: 31.0725; **Identification:** identifiedBy: M. El-Hawagry; dateIdentified: May 2020; **Event:** samplingProtocol: Sweeping; eventDate: 08-22-1925; **Record Level:** institutionCode: EFC
- **Type status:** Other material. **Occurrence:** recordedBy: Farag; sex: 1 female; lifeStage: adult; **Taxon:** taxonID: https://www.gbif.org/species/5076777; scientificName: Conops elegans; kingdom: Animalia; phylum: Arthropoda; class: Insecta; order: Diptera; family: Conopidae; **Location:** country: Egypt; locality: Mansouriah; decimalLatitude: 30.1236; decimalLongitude: 31.0725; **Identification:** identifiedBy: M. El-Hawagry; dateIdentified: May 2020; **Event:** samplingProtocol: Sweeping; eventDate: 05-12-1926; **Record Level:** institutionCode: EFC
- **Type status:** Other material. **Occurrence:** recordedBy: H.C.E & M.T.; sex: 1 female; lifeStage: adult; **Taxon:** taxonID: https://www.gbif.org/species/5076777; scientificName: Conops elegans; kingdom: Animalia; phylum: Arthropoda; class: Insecta; order: Diptera; family: Conopidae; **Location:** country: Egypt; locality: Mansouriah; decimalLatitude: 30.1236; decimalLongitude: 31.0725; **Identification:** identifiedBy: M. El-Hawagry; dateIdentified: May 2020; **Event:** samplingProtocol: Sweeping; eventDate: 05-12-1926; **Record Level:** institutionCode: EFC
- **Type status:** Other material. **Occurrence:** recordedBy: R.M.; sex: 1 male; lifeStage: adult; **Taxon:** taxonID: https://www.gbif.org/species/5076777; scientificName: Conops elegans; kingdom: Animalia; phylum: Arthropoda; class: Insecta; order: Diptera; family: Conopidae; **Location:** country: Egypt; locality: Mansouriah; decimalLatitude: 30.1236; decimalLongitude: 31.0725; **Identification:** identifiedBy: M. El-Hawagry; dateIdentified: May 2020; **Event:** samplingProtocol: Sweeping; eventDate: 04-26-1930; **Record Level:** institutionCode: EFC
- **Type status:** Other material. **Occurrence:** recordedBy: Shafik; sex: 2 females; lifeStage: adult; **Taxon:** taxonID: https://www.gbif.org/species/5076777; scientificName: Conops elegans; kingdom: Animalia; phylum: Arthropoda; class: Insecta; order: Diptera; family: Conopidae; **Location:** country: Egypt; locality: Rafah; decimalLatitude: 31.2667; decimalLongitude: 34.2333; **Identification:** identifiedBy: M. El-Hawagry; dateIdentified: May 2020; **Event:** samplingProtocol: Sweeping; eventDate: 05-17-1951; **Record Level:** institutionCode: EFC
- **Type status:** Other material. **Occurrence:** sex: 1 female; lifeStage: adult; **Taxon:** taxonID: https://www.gbif.org/species/5076777; scientificName: Conops elegans; kingdom: Animalia; phylum: Arthropoda; class: Insecta; order: Diptera; family: Conopidae; **Location:** country: Egypt; locality: Sakkara; decimalLatitude: 29.849552; decimalLongitude: 31.2164; **Identification:** identifiedBy: M. El-Hawagry; dateIdentified: May 2020; **Event:** samplingProtocol: Sweeping; eventDate: 06-08-1917; **Record Level:** institutionCode: EFC
- **Type status:** Other material. **Occurrence:** recordedBy: El-Hawagry; sex: 1 male, 1 female; lifeStage: adult; **Taxon:** taxonID: https://www.gbif.org/species/5076777; scientificName: Conops elegans; kingdom: Animalia; phylum: Arthropoda; class: Insecta; order: Diptera; family: Conopidae; **Location:** country: Saudi Arabia; locality: Al-Mekhwa; decimalLatitude: 19.8429; decimalLongitude: 41.3115; **Identification:** identifiedBy: M. El-Hawagry; dateIdentified: May 2020; **Event:** samplingProtocol: Sweeping; eventDate: 03-17-1912; **Record Level:** institutionCode: MSHC

##### Distribution

AF: Democratic Republic of the Congo, Ethiopia, Gabon, Guinea, Guinea-Bissau, Malawi, Mozambique, Namibia, Nigeria, Saudi Arabia [as “South-western part”] (first record), South Africa, Tanzania, Yemen. PA: Algeria, Bulgaria, Cyprus, Egypt, France, Israel, Italy, Morocco, Romania, Spain, Syria, Tunisia, Turkey.

**Figure 2.**
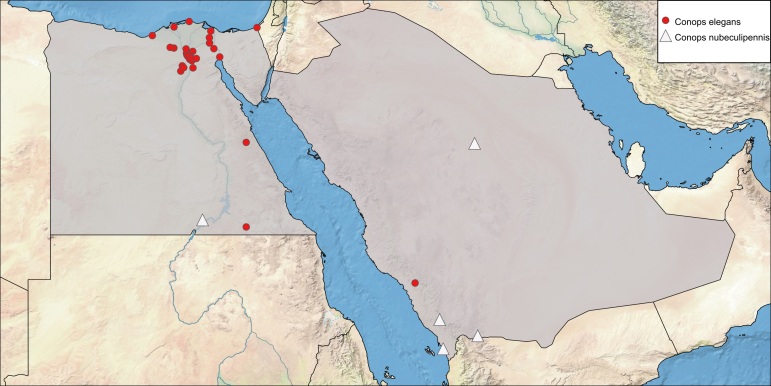
Local distribution map of Conops (Asiconops) elegans Meigen and Conops (Asiconops) nubeculipennis Bezzi in Egypt and Saudi Arabia.

**Local distribution and dates of collection** (Fig. 2): EGYPT: Coastal Strip: Balteem, Burg (March to August); Eastern Desert: Abu-Sueir, El-Mallah (= Mallah East), Fayed, Suez, Wadi Garawi, Wadi Hoff, Wadi Morrah, Wadi Rishrash, Wadi Um-Elek, Wadi Zohleiga (March to August); Fayoum: Fayoum City, Kom Osheem, Tamiya (May to November); Lower Nile Valley and Delta: Abu-Rawash, Barrage, Edfina, El-Gebel El-Asfar, El-Magadlah, Ezbet El-Nakhl, Gezira, Giza, Helwan, Kafr Ghatati, Kafr Hakim, Kerdassa, Maadi, Mansouriah, Orman, Pyramids, Sentris (throughout the year); Sinai: Rafah (August); Western Desert: Saqqara, Madinet El-Sadat, Wadi El-Natroun (June and October) [Sources: Kröber (1927), Azmy (2016) and museum material in EFC and ESEC]. SAUDI ARABIA: Al-Baha: Al-Mekhwa (March) [Sources: collected material].

##### Notes

This species (Fig. 12a) is recorded herein for the first time from Saudi Arabia.

#### Conops (Asiconops) flavifrons

Meigen, 1804

D4A808AA-1F11-533D-B289-291E663950AC

https://www.gbif.org/species/5076765

Conops flavifrons Meigen, 1804: 281. Type locality: France (Lyon).
Physocephala pugioniformis Becker, 1913: 611. Type locality: Iran.
Conops minor Becker, 1922: 201. Type locality: Russia, Italy, Hungary & Greece.
Conops kroeberi var. immaculata Paramonov, 1927: 4. Type locality: Armenia.
Conops kroeberi Paramonov, 1927: 4. Type locality: Armenia.

##### Distribution

AF: Yemen. PA: Albania, Armenia, Austria, ?Belgium, Bulgaria, ?China, Croatia, Czech Republic, Egypt, France, ?Germany, Greece, Hungary, Iran, Italy, Poland, Russia, Serbia, Spain, Syria, Tunisia, Turkey.

##### Notes

This species was listed by Steyskal and El-Bialy (1967), Chvála and Smith (1988), Azmy (2016) as recorded from Egypt, but these records could not be verified and require confirmation as no specimens have been collected or preserved in Egyptian museums.

#### Conops (Asiconops) nubeculipennis

Bezzi, 1901

8C73F907-C774-50D8-964C-66C249D68A06

https://www.gbif.org/species/5076815

Conops nubeculipennis Bezzi, 1901: 21. Type locality: Eritrea.
Conops atrogonius Séguy 1930: 134. Type locality: Morocco.

##### Materials

- **Type status:** Other material. **Occurrence:** recordedBy: Aldhafer H.M. et al.; sex: 1 male; lifeStage: adult; **Taxon:** taxonID: https://www.gbif.org/species/5076815; scientificName: Conops nubeculipennis; kingdom: Animalia; phylum: Arthropoda; class: Insecta; order: Diptera; family: Conopidae; **Location:** country: Saudi Arabia; stateProvince: Al-Qassim; locality: Unayzah; decimalLatitude: 26.085478; decimalLongitude: 43.9768123; **Identification:** identifiedBy: M. El-Hawagry; dateIdentified: May 2020; **Event:** samplingProtocol: Malaise trap; eventDate: 05-01-2018; **Record Level:** institutionCode: KSMA
- **Type status:** Other material. **Occurrence:** recordedBy: Aldhafer H.M. et al.; sex: 1 female; lifeStage: adult; **Taxon:** taxonID: https://www.gbif.org/species/5076815; scientificName: Conops nubeculipennis; kingdom: Animalia; phylum: Arthropoda; class: Insecta; order: Diptera; family: Conopidae; **Location:** country: Saudi Arabia; stateProvince: Asir; locality: Garf Raydah Nature Reserve; decimalLatitude: 18.194917; decimalLongitude: 42.396967; **Identification:** identifiedBy: M. El-Hawagry; dateIdentified: May 2020; **Event:** samplingProtocol: Malaise trap; eventDate: 05-08-2015; **Record Level:** institutionCode: KSMA

##### Distribution

AF: Cameroon, Ethiopia, Guinea-Bissau, Mozambique, Nigeria, Saudi Arabia [as “South-western part”] (El-Hawagry et al. 2017), Yemen, Zambia. PA: Egypt, Israel, Morocco.

**Local distribution and dates of collection** (Fig. 2): EGYPT: Upper Nile Valley: South Aswan (as “Nubien”) [Source: Kröber (1915b)]. SAUDI ARABIA: Al-Qassim: Unayzah (May); Asir: Raydah Nature Reserve (May and June); South-western part of Saudi Arabia including Jazan and Najran (September and October) [Sources: Walker and Pittaway (1987), El-Hawagry et al. (2017)) and collected material].

##### Notes

This species was listed by Kröber (1915b), Steyskal and El-Bialy (1967), Azmy (2016), Stuke (2017) as recorded from Egypt, but no specimens have been collected or preserved in Egyptian museums.

#### Conops

Linnaeus

95513370-78CD-5EE7-B198-0A8838230483

#### Conops (Conops) flavicauda

(Bigot, 1880)

C37A3C82-52E9-5907-A837-5A5656702F6D

https://www.gbif.org/species/5076720

Sphixosoma flavicauda Bigot, 1880: 149. Type locality: Iran (Northern Iran and Caucasus).
Conops euzonatus Bigot, 1887: 33. Type locality: Caucasus Mountains.
Conops superbus Röder, 1889: 6. Type locality: Lebanon (Beirut) [as “Beyrut, Syria (Asia minor)”].
Conops cypris Janssens, 1955: 2. Type locality: Cyprus (Akrotiri Bay, Yermdoyia & Erimi).

##### Distribution

AF: Uganda. PA: Afghanistan, Armenia, Cyprus, Egypt, Greece, Iran, Israel, Lebanon, Syria, Turkey.

**Figure 3.**
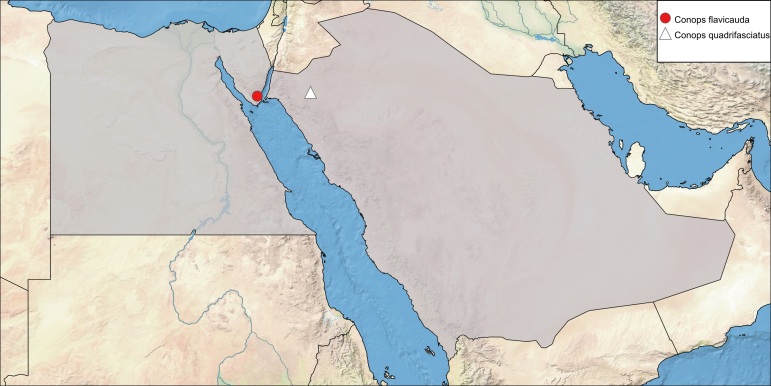
Local distribution map of Conops (Conops) flavicauda (Bigot) and Conops (Conops) quadrifasciatus De Geer in Egypt and Saudi Arabia.

**Local distribution and dates of collection** (Fig. 3): EGYPT: Sinai: Wadi Isla (August) [Sources: Zalat et al. 2008].

#### Conops (Conops) quadrifasciatus

De Geer, 1776

527F63CF-C9C2-5220-AFC6-5EBFE9727F0E

https://www.gbif.org/species/5076797

Conops De Geer, 1776: 261. Type locality: Presumably Sweden.

##### Materials

- **Type status:** Other material. **Occurrence:** recordedBy: El-Hawagry; sex: 1 male; lifeStage: adult; **Taxon:** taxonID: https://www.gbif.org/species/5076797; scientificName: Conops quadrifasciatus; kingdom: Animalia; phylum: Arthropoda; class: Insecta; order: Diptera; family: Conopidae; **Location:** country: Saudi Arabia; stateProvince: Tabouk; locality: Tabouk; decimalLatitude: 28.36661; decimalLongitude: 36.629747; **Identification:** identifiedBy: M. El-Hawagry; dateIdentified: May 2020; **Event:** samplingProtocol: Malaise trap; eventDate: 10-27-2014; **Record Level:** institutionCode: MSHC

##### Distribution

PA: Andorra, Austria, Belarus, Belgium, Bosnia and Herzegovina, Bulgaria, Croatia, Czech Republic, Denmark, Finland, France, Germany, Great Britain, Hungary, Iran, Ireland, Italy, Kazakhstan, Latvia, Lithuania, Netherlands, Norway, Poland, Romania, Russia, Saudi Arabia (first record), Slovakia, Spain, Sweden, Switzerland, Turkey, Ukraine.

**Local distribution and dates of collection** (Fig. 3): SAUDI ARABIA: Tabouk (October) [Source: collected material].

##### Notes

This species is recorded herein for the first time from Saudi Arabia.

#### Conops (Conops) rufiventris

Macquart, 1849

0BBA3D38-99F8-55E7-8D52-4013A9F89E3B

https://www.gbif.org/species/5076705

Conops rufiventris Macquart, 1849: 474. Type locality: Algeria (Constantine).

##### Materials

- **Type status:** Other material. **Occurrence:** recordedBy: Willson; sex: 1 male; lifeStage: adult; **Taxon:** taxonID: https://www.gbif.org/species/5076705; scientificName: Conops rufiventris; kingdom: Animalia; phylum: Arthropoda; class: Insecta; order: Diptera; family: Conopidae; **Location:** country: Egypt; locality: Salloum; decimalLatitude: 31.588751; decimalLongitude: 25.102649; **Identification:** identifiedBy: M. El-Hawagry; dateIdentified: May 2020; **Event:** samplingProtocol: Sweeping; eventDate: 05-14-1917; **Record Level:** institutionCode: EFC

##### Distribution

Algeria, Egypt, Tunisia.

**Figure 4.**
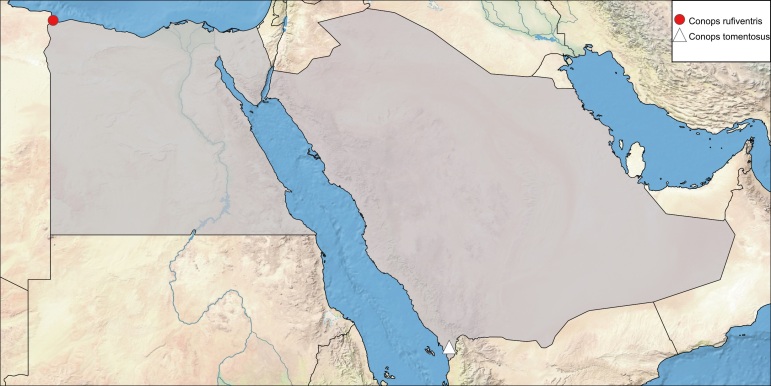
Local distribution map of Conops (Conops) rufiventris Macquart and Conops (Conops) tomentosus Kröber in Egypt and Saudi Arabia.

**Local distribution and dates of collection** (Fig. 4): EGYPT: Coastal Strip: Salloum (April and May) [Azmy (2016) and museum material in EFC].

#### Conops (Conops) tomentosus

Kröber, 1916

C35CEF87-DDD3-53A9-BB2B-012315BB75EC

https://www.gbif.org/species/5076849

Conops tomentosus Kröber, 1916: 45. Type locality: Turkmenistan.

##### Distribution

AF: Saudi Arabia [as “South-western part”]. PA: Iraq, Tajikistan, Turkey, Turkmenistan.

**Local distribution and dates of collection** (Fig. 4): SAUDI ARABIA: Jazan: Abu-Arish (March) [Source: Stuke et al. (2008)].

#### PHYSOCEPHALINI

0CF0A37A-3709-53B4-8806-EF4E4B18A3A7

#### Physocephala

Schiner

2ABD2B29-4CB6-56BF-B851-931FE8B51E63

https://www.gbif.org/species/1568868

Physocephala ***Physocephala*** Schiner, 1861: 138. Type species: *Conops rufipes* Fabricius, by original designation.

#### Physocephala antiqua

(Wiedemann, 1830)

F05F8C8C-6F7A-539B-8DE6-96B58ED73222

https://www.gbif.org/species/1569080

Conops antiqua Wiedemann, 1830: 239. Type locality: Egypt.
Conops arabica Macquart, 1851: 134. Type locality: Saudi Arabia (Jeddah).
Physocephala furax Becker, 1913: 612. Type locality: Iran.
Physocephala syriaca Kröber, 1915: 72. Type locality: Egypt (Cairo) & Palestinian West Bank (Jericho).
Physocephala obscurifacies Kröber, 1924: 67. Type locality: Egypt (Wadi Hoff).

##### Materials

- **Type status:** Other material. **Occurrence:** recordedBy: Tewfik; sex: 1 female; lifeStage: adult; **Taxon:** taxonID: https://www.gbif.org/species/1569080; scientificName: Physocephala antiqua; kingdom: Animalia; phylum: Arthropoda; class: Insecta; order: Diptera; family: Conopidae; **Location:** country: Egypt; locality: Abu-Sueir; decimalLatitude: 30.5766; decimalLongitude: 32.107622; **Identification:** identifiedBy: M. El-Hawagry; dateIdentified: May 2020; **Event:** samplingProtocol: Sweeping; eventDate: 05-17-1925; **Record Level:** institutionCode: EFC
- **Type status:** Other material. **Occurrence:** recordedBy: H.C.E & M.T.; sex: 1 female; lifeStage: adult; **Taxon:** taxonID: https://www.gbif.org/species/1569080; scientificName: Physocephala antiqua; kingdom: Animalia; phylum: Arthropoda; class: Insecta; order: Diptera; family: Conopidae; **Location:** country: Egypt; locality: Dar El-Maskhara; decimalLatitude: 29.7833; decimalLongitude: 31.4167; **Identification:** identifiedBy: M. El-Hawagry; dateIdentified: May 2020; **Event:** samplingProtocol: Sweeping; eventDate: 04-11-1927; **Record Level:** collectionCode: EFC
- **Type status:** Other material. **Occurrence:** recordedBy: R.M.; sex: 1 male; lifeStage: adult; **Taxon:** taxonID: https://www.gbif.org/species/1569080; scientificName: Physocephala antiqua; kingdom: Animalia; phylum: Arthropoda; class: Insecta; order: Diptera; family: Conopidae; **Location:** country: Egypt; locality: El-Mallah; decimalLatitude: 30.8167; decimalLongitude: 32.1000; **Identification:** identifiedBy: M. El-Hawagry; dateIdentified: May 2020; **Event:** samplingProtocol: Sweeping; eventDate: 04-29-1927; **Record Level:** institutionCode: EFC
- **Type status:** Other material. **Occurrence:** recordedBy: H.C.E & M.T.; sex: 1 male; lifeStage: adult; **Taxon:** taxonID: https://www.gbif.org/species/1569080; scientificName: Physocephala antiqua; kingdom: Animalia; phylum: Arthropoda; class: Insecta; order: Diptera; family: Conopidae; **Location:** country: Egypt; locality: W. Garawi; decimalLatitude: 30.7833; decimalLongitude: 31.3167; **Identification:** identifiedBy: M. El-Hawagry; dateIdentified: May 2020; **Event:** samplingProtocol: Sweeping; eventDate: 05-15-1927; **Record Level:** institutionCode: EFC
- **Type status:** Other material. **Occurrence:** recordedBy: Farag; sex: 1 male; lifeStage: adult; **Taxon:** taxonID: https://www.gbif.org/species/1569080; scientificName: Physocephala antiqua; kingdom: Animalia; phylum: Arthropoda; class: Insecta; order: Diptera; family: Conopidae; **Location:** country: Egypt; locality: Wadi Digla; decimalLatitude: 29.9578; decimalLongitude: 31.3348; **Identification:** identifiedBy: M. El-Hawagry; dateIdentified: May 2020; **Event:** samplingProtocol: Sweeping; eventDate: 09-03-1926; **Record Level:** institutionCode: EFC
- **Type status:** Other material. **Occurrence:** recordedBy: Tewfik; sex: 1 male; lifeStage: adult; **Taxon:** taxonID: https://www.gbif.org/species/1569080; scientificName: Physocephala antiqua; kingdom: Animalia; phylum: Arthropoda; class: Insecta; order: Diptera; family: Conopidae; **Location:** country: Egypt; locality: Wadi Rishrash; decimalLatitude: 29.4642; decimalLongitude: 31.3672; **Identification:** identifiedBy: M. El-Hawagry; dateIdentified: May 2020; **Event:** samplingProtocol: Sweeping; eventDate: 06-17-1932; **Record Level:** institutionCode: EFC
- **Type status:** Other material. **Occurrence:** recordedBy: R.M.; sex: 1 male; lifeStage: adult; **Taxon:** taxonID: https://www.gbif.org/species/1569080; scientificName: Physocephala antiqua; kingdom: Animalia; phylum: Arthropoda; class: Insecta; order: Diptera; family: Conopidae; **Location:** country: Egypt; locality: Abu-Rawash; decimalLatitude: 30.043845; decimalLongitude: 31.092932; **Identification:** identifiedBy: M. El-Hawagry; dateIdentified: May 2020; **Event:** samplingProtocol: Sweeping; eventDate: 08-15-1925; **Record Level:** institutionCode: EFC
- **Type status:** Other material. **Occurrence:** recordedBy: R.M.; sex: 2 males; lifeStage: adult; **Taxon:** taxonID: https://www.gbif.org/species/1569080; scientificName: Physocephala antiqua; kingdom: Animalia; phylum: Arthropoda; class: Insecta; order: Diptera; family: Conopidae; **Location:** country: Egypt; locality: Abu-Rawash; decimalLatitude: 30.043845; decimalLongitude: 31.092932; **Identification:** identifiedBy: M. El-Hawagry; dateIdentified: May 2020; **Event:** samplingProtocol: Sweeping; eventDate: 07-18-1925; **Record Level:** institutionCode: EFC
- **Type status:** Other material. **Occurrence:** recordedBy: R.M.; sex: 1 male; lifeStage: adult; **Taxon:** taxonID: https://www.gbif.org/species/1569080; scientificName: Physocephala antiqua; kingdom: Animalia; phylum: Arthropoda; class: Insecta; order: Diptera; family: Conopidae; **Location:** country: Egypt; locality: Abu-Rawash; decimalLatitude: 30.043845; decimalLongitude: 31.092932; **Identification:** identifiedBy: M. El-Hawagry; dateIdentified: May 2020; **Event:** samplingProtocol: Sweeping; eventDate: 06-06-1925; **Record Level:** institutionCode: EFC
- **Type status:** Other material. **Occurrence:** recordedBy: Farag; sex: 1 male; lifeStage: adult; **Taxon:** taxonID: https://www.gbif.org/species/1569080; scientificName: Physocephala antiqua; kingdom: Animalia; phylum: Arthropoda; class: Insecta; order: Diptera; family: Conopidae; **Location:** country: Egypt; locality: Helwan; decimalLatitude: 29.8500; decimalLongitude: 31.3333; **Identification:** identifiedBy: M. El-Hawagry; dateIdentified: May 2020; **Event:** samplingProtocol: Sweeping; eventDate: 04-23-1935; **Record Level:** institutionCode: EFC
- **Type status:** Other material. **Occurrence:** recordedBy: R.M.; sex: 2 males; lifeStage: adult; **Taxon:** taxonID: https://www.gbif.org/species/1569080; scientificName: Physocephala antiqua; kingdom: Animalia; phylum: Arthropoda; class: Insecta; order: Diptera; family: Conopidae; **Location:** country: Egypt; locality: Kerdassa; decimalLatitude: 30.0297; decimalLongitude: 31.1061; **Identification:** identifiedBy: M. El-Hawagry; dateIdentified: May 2020; **Event:** samplingProtocol: Sweeping; eventDate: 04-17-1926; **Record Level:** institutionCode: EFC
- **Type status:** Other material. **Occurrence:** recordedBy: R.M.; sex: 2 males; lifeStage: adult; **Taxon:** taxonID: https://www.gbif.org/species/1569080; scientificName: Physocephala antiqua; kingdom: Animalia; phylum: Arthropoda; class: Insecta; order: Diptera; family: Conopidae; **Location:** country: Egypt; locality: Mansouriah; decimalLatitude: 30.1236; decimalLongitude: 31.07246; **Identification:** identifiedBy: M. El-Hawagry; dateIdentified: May 2020; **Event:** samplingProtocol: Sweeping; eventDate: 09-02-1925; **Record Level:** institutionCode: EFC
- **Type status:** Other material. **Occurrence:** recordedBy: El-Hawagry; sex: 1 male; lifeStage: adult; **Taxon:** taxonID: https://www.gbif.org/species/1569080; scientificName: Physocephala antiqua; kingdom: Animalia; phylum: Arthropoda; class: Insecta; order: Diptera; family: Conopidae; **Location:** country: Saudi Arabia; stateProvince: Al-Baha; locality: Al-Mekhwa; decimalLatitude: 19.8429; decimalLongitude: 41.3115; **Identification:** identifiedBy: M. El-Hawagry; dateIdentified: May 2020; **Event:** samplingProtocol: Sweeping; eventDate: 03-17-2012; **Record Level:** institutionCode: MSHC
- **Type status:** Other material. **Occurrence:** recordedBy: Aldhafer H.M. et al.; sex: 1 male; lifeStage: adult; **Taxon:** taxonID: https://www.gbif.org/species/1569080; scientificName: Physocephala antiqua; kingdom: Animalia; phylum: Arthropoda; class: Insecta; order: Diptera; family: Conopidae; **Location:** country: Saudi Arabia; stateProvince: Jazan; locality: Jazan; decimalLatitude: 16.902448; decimalLongitude: 42.570516; **Identification:** identifiedBy: M. El-Hawagry; dateIdentified: May 2020; **Event:** samplingProtocol: Malaise trap; eventDate: 05-12-2019; **Record Level:** institutionCode: KSMA

##### Distribution

AF: Egypt [as “Gebel Elba”], Saudi Arabia [as “South-western part”], United Arab Emirates, Yemen. PA: Afghanistan, Algeria, China, Cyprus, Egypt, Greece, Iran, Iraq, Israel, Italy, Jordan, Kazakhstan, Kyrgyzstan, Mongolia, Palestinian Territories, Russia, Saudi Arabia (Jeddah), Syria, Tunisia, Turkey, Turkmenistan.

**Figure 5.**
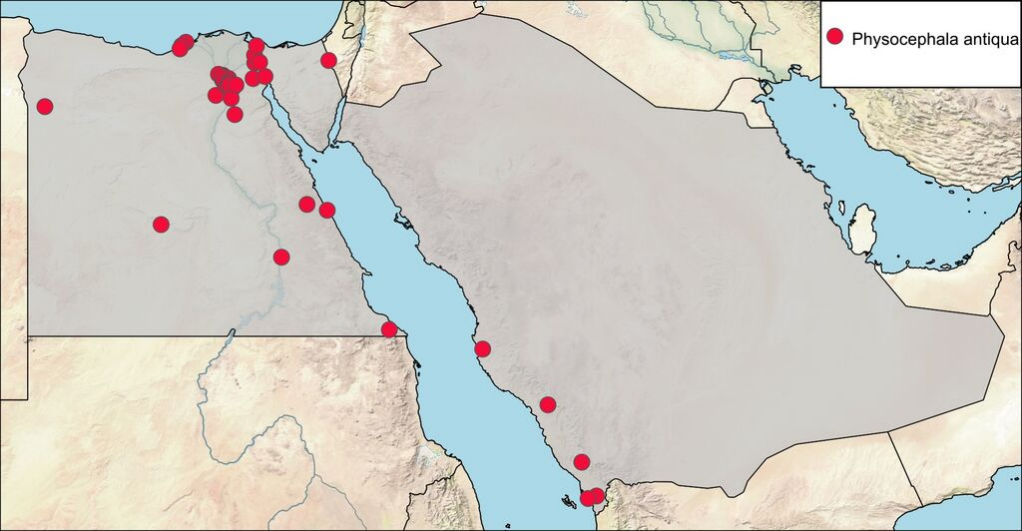
Local distribution map of *Physocephala antiqua* (Wiedemann) in Egypt and Saudi Arabia.

**Local distribution and dates of collection** (Fig. 5): EGYPT: Coastal Strip: Abu-Kir, Dekhela, Mariout (April to August); Eastern Desert: Abu Sueir, Cairo-Suez Road, El-Mallah, Geneifa, Ismailia, Wadi Asal (30 km S. Kosseir), Wadi Digla, Wadi El-Maskhara, Wadi Garawi, Wadi Gharagid, Wadi Hoff, Wadi Ibtadi, Wadi Rishrash (February to September); Fayoum: Kom Osheem (May, August and November); Gebel Elba: locality unknown (January); Lower Nile Valley and Delta: Abu-Rawash, Barrage, Burgash, Cairo, El-Katta, El-Magadlah, Ezbet El-Nakhl, Helwan, Kafr Hakim, Kerdassa, Maadi, Mansouriah, Pyramids, Turah (April to October); Sinai: Wadi Gedeirat (June and July); Upper Nile Valley: Kom Ombo (January); Western Desert: Dahshour, Dakhla Oasis (Balat, Ewina & Rashda), Saqqara, Siwa Oasis (March to December) [Sources: Kröber (1915a), Kröber (1924), Kröber (1927), Stuke and Clements (2008), Stuke (2014), Azmy (2016) and museum material in EFC and ESEC]. SAUDI ARABIA: Al-Baha: Al-Mekhwa (March); Asir: Marabah (April); Jazan: Abu-Arish Road (Al-Mahdag and Arada), Jazan City (March to May); Makka Al-Mukarramah: Jeddah (date unknown) [Sources: Kröber (1915a), Stuke and Clements (2008) and collected material].

#### Physocephala chrysorrhoea

(Meigen, 1824)

9F4DA306-0CC6-5601-8A31-9FE15AF9280D

https://www.gbif.org/species/1569093

Conops chrysorrhoea Meigen, 1824: 128. Type locality: Austria.
Conops pallasi Meigen, 1824: 128. Type locality: Russia.
Conops serpylleti Zeller, 1842: 837. Type locality: Germany.
Physocephala zarudnyi Becker, 1913: 614. Type locality: Iran.
Physocephala truncata var. pseudomaculigera Kröber, 1915: 72. Type locality: Egypt & Tunisia.

##### Materials

- **Type status:** Other material. **Occurrence:** recordedBy: H.C.E & M.T.; sex: 1 female; lifeStage: adult; **Taxon:** taxonID: https://www.gbif.org/species/1569093; scientificName: Physocephala chrysorrhoea; kingdom: Animalia; phylum: Arthropoda; class: Insecta; order: Diptera; family: Conopidae; **Location:** country: Egypt; locality: Burg; decimalLatitude: 30.9081; decimalLongitude: 29.5464; **Identification:** identifiedBy: M. El-Hawagry; dateIdentified: June 2020; **Event:** samplingProtocol: Sweeping; eventDate: 03-25-1927; **Record Level:** institutionCode: EFC
- **Type status:** Other material. **Occurrence:** recordedBy: Tewfik; sex: 1 male, 1 female; lifeStage: adult; **Taxon:** taxonID: https://www.gbif.org/species/1569093; scientificName: Physocephala chrysorrhoea; kingdom: Animalia; phylum: Arthropoda; class: Insecta; order: Diptera; family: Conopidae; **Location:** country: Egypt; locality: Burg; decimalLatitude: 30.9081; decimalLongitude: 29.5464; **Identification:** identifiedBy: M. El-Hawagry; dateIdentified: June 2020; **Event:** samplingProtocol: Sweeping; eventDate: 05-10-1927; **Record Level:** institutionCode: EFC
- **Type status:** Other material. **Occurrence:** recordedBy: Selim; sex: 1 male; lifeStage: adult; **Taxon:** taxonID: https://www.gbif.org/species/1569093; scientificName: Physocephala chrysorrhoea; kingdom: Animalia; phylum: Arthropoda; class: Insecta; order: Diptera; family: Conopidae; **Location:** country: Egypt; locality: Ein Moussa; decimalLatitude: 29.8667; decimalLongitude: 32.6500; **Identification:** identifiedBy: M. El-Hawagry; dateIdentified: June 2020; **Event:** samplingProtocol: Sweeping; eventDate: 02-07-1928; **Record Level:** institutionCode: ESEC
- **Type status:** Other material. **Occurrence:** recordedBy: Farag; sex: 1 female; lifeStage: adult; **Taxon:** taxonID: https://www.gbif.org/species/1569093; scientificName: Physocephala chrysorrhoea; kingdom: Animalia; phylum: Arthropoda; class: Insecta; order: Diptera; family: Conopidae; **Location:** country: Egypt; locality: W. Dar El-Maskhara; decimalLatitude: 29.7833; decimalLongitude: 31.4167; **Identification:** identifiedBy: M. El-Hawagry; dateIdentified: June 2020; **Event:** samplingProtocol: Sweeping; eventDate: 03-21-1927; **Record Level:** institutionCode: EFC
- **Type status:** Other material. **Occurrence:** recordedBy: H.C.E & M.T.; sex: 1 female; lifeStage: adult; **Taxon:** taxonID: https://www.gbif.org/species/1569093; scientificName: Physocephala chrysorrhoea; kingdom: Animalia; phylum: Arthropoda; class: Insecta; order: Diptera; family: Conopidae; **Location:** country: Egypt; locality: W. Garawi; decimalLatitude: 29.7833; decimalLongitude: 31.3167; **Identification:** identifiedBy: M. El-Hawagry; dateIdentified: June 2020; **Event:** samplingProtocol: Sweeping; eventDate: 03-25-1932; **Record Level:** institutionCode: EFC
- **Type status:** Other material. **Occurrence:** recordedBy: H.C.E & M.T.; sex: 2 males; lifeStage: adult; **Taxon:** taxonID: https://www.gbif.org/species/1569093; scientificName: Physocephala chrysorrhoea; kingdom: Animalia; phylum: Arthropoda; class: Insecta; order: Diptera; family: Conopidae; **Location:** country: Egypt; locality: W. Morrah; decimalLatitude: 22.3500; decimalLongitude: 33.7500; **Identification:** identifiedBy: M. El-Hawagry; dateIdentified: June 2020; **Event:** samplingProtocol: Sweeping; eventDate: 03-01-1925; **Record Level:** institutionCode: EFC
- **Type status:** Other material. **Occurrence:** recordedBy: Farag; sex: 1 male; lifeStage: adult; **Taxon:** taxonID: https://www.gbif.org/species/1569093; scientificName: Physocephala chrysorrhoea; kingdom: Animalia; phylum: Arthropoda; class: Insecta; order: Diptera; family: Conopidae; **Location:** country: Egypt; locality: Wadi Hoff; decimalLatitude: 29.8820558; decimalLongitude: 31.3109855; **Identification:** identifiedBy: M. El-Hawagry; dateIdentified: June 2020; **Event:** samplingProtocol: Sweeping; eventDate: 03-04-1927; **Record Level:** institutionCode: EFC
- **Type status:** Other material. **Occurrence:** recordedBy: Efflatoun; sex: 1 male; lifeStage: adult; **Taxon:** taxonID: https://www.gbif.org/species/1569093; scientificName: Physocephala chrysorrhoea; kingdom: Animalia; phylum: Arthropoda; class: Insecta; order: Diptera; family: Conopidae; **Location:** country: Egypt; locality: Wadi Um-Elek; decimalLatitude: 29.8833; decimalLongitude: 31.5167; **Identification:** identifiedBy: M. El-Hawagry; dateIdentified: June 2020; **Event:** samplingProtocol: Sweeping; eventDate: 03-12-1924; **Record Level:** institutionCode: EFC
- **Type status:** Other material. **Occurrence:** recordedBy: Farag; sex: 1 male; lifeStage: adult; **Taxon:** taxonID: https://www.gbif.org/species/1569093; scientificName: Physocephala chrysorrhoea; kingdom: Animalia; phylum: Arthropoda; class: Insecta; order: Diptera; family: Conopidae; **Location:** country: Egypt; locality: Wadi Um-Elek; decimalLatitude: 29.8833; decimalLongitude: 31.5167; **Identification:** identifiedBy: M. El-Hawagry; dateIdentified: June 2020; **Event:** samplingProtocol: Sweeping; eventDate: 03-13-1930; **Record Level:** institutionCode: EFC
- **Type status:** Other material. **Occurrence:** recordedBy: Shafik & Str.EFC; sex: 1 male; lifeStage: adult; **Taxon:** taxonID: https://www.gbif.org/species/1569093; scientificName: Physocephala chrysorrhoea; kingdom: Animalia; phylum: Arthropoda; class: Insecta; order: Diptera; family: Conopidae; **Location:** country: Egypt; locality: Girza; decimalLatitude: 29.499684; decimalLongitude: 31.073799; **Identification:** identifiedBy: M. El-Hawagry; dateIdentified: June 2020; **Event:** samplingProtocol: Sweeping; eventDate: 03-05-1950; **Record Level:** institutionCode: EFC
- **Type status:** Other material. **Occurrence:** recordedBy: Farag; sex: 1 male; lifeStage: adult; **Taxon:** taxonID: https://www.gbif.org/species/1569093; scientificName: Physocephala chrysorrhoea; kingdom: Animalia; phylum: Arthropoda; class: Insecta; order: Diptera; family: Conopidae; **Location:** country: Egypt; locality: Abu-Rawash; decimalLatitude: 30.043845; decimalLongitude: 31.092932; **Identification:** identifiedBy: M. El-Hawagry; dateIdentified: June 2020; **Event:** samplingProtocol: Sweeping; eventDate: 05-05-1926; **Record Level:** institutionCode: EFC
- **Type status:** Other material. **Occurrence:** recordedBy: R.M.; sex: 1 female; lifeStage: adult; **Taxon:** taxonID: https://www.gbif.org/species/1569093; scientificName: Physocephala chrysorrhoea; kingdom: Animalia; phylum: Arthropoda; class: Insecta; order: Diptera; family: Conopidae; **Location:** country: Egypt; locality: Abu-Rawash; decimalLatitude: 30.043845; decimalLongitude: 31.092932; **Identification:** identifiedBy: M. El-Hawagry; dateIdentified: June 2020; **Event:** samplingProtocol: Sweeping; eventDate: 04-03-1926; **Record Level:** institutionCode: EFC
- **Type status:** Other material. **Occurrence:** recordedBy: Farag; sex: 3 females; lifeStage: adult; **Taxon:** taxonID: https://www.gbif.org/species/1569093; scientificName: Physocephala chrysorrhoea; kingdom: Animalia; phylum: Arthropoda; class: Insecta; order: Diptera; family: Conopidae; **Location:** country: Egypt; locality: Helwan; decimalLatitude: 29.8500; decimalLongitude: 31.3333; **Identification:** identifiedBy: M. El-Hawagry; dateIdentified: June 2020; **Event:** samplingProtocol: Sweeping; eventDate: 04-13-1935; **Record Level:** institutionCode: EFC
- **Type status:** Other material. **Occurrence:** recordedBy: Farag; sex: 1 male, 2 females; lifeStage: adult; **Taxon:** taxonID: https://www.gbif.org/species/1569093; scientificName: Physocephala chrysorrhoea; kingdom: Animalia; phylum: Arthropoda; class: Insecta; order: Diptera; family: Conopidae; **Location:** country: Egypt; locality: Helwan; decimalLatitude: 29.8500; decimalLongitude: 31.3333; **Identification:** identifiedBy: M. El-Hawagry; dateIdentified: June 2020; **Event:** samplingProtocol: Sweeping; eventDate: 04-28-1934; **Record Level:** institutionCode: EFC
- **Type status:** Other material. **Occurrence:** recordedBy: Shafik; sex: 3 males, 3 females; lifeStage: adult; **Taxon:** taxonID: https://www.gbif.org/species/1569093; scientificName: Physocephala chrysorrhoea; kingdom: Animalia; phylum: Arthropoda; class: Insecta; order: Diptera; family: Conopidae; **Location:** country: Egypt; locality: Ein Gedeirat; decimalLatitude: 30.6500; decimalLongitude: 34.4333; **Identification:** identifiedBy: M. El-Hawagry; dateIdentified: June 2020; **Event:** samplingProtocol: Sweeping; eventDate: 5/24-29/1938; **Record Level:** institutionCode: EFC
- **Type status:** Other material. **Occurrence:** recordedBy: H.C.E & M.T.; sex: 2 males, 1 female; lifeStage: adult; **Taxon:** taxonID: https://www.gbif.org/species/1569093; scientificName: Physocephala chrysorrhoea; kingdom: Animalia; phylum: Arthropoda; class: Insecta; order: Diptera; family: Conopidae; **Location:** country: Egypt; locality: W. El-Arbaein (Sinai); decimalLatitude: 28.5469; decimalLongitude: 33.9530; **Identification:** identifiedBy: M. El-Hawagry; dateIdentified: June 2020; **Event:** samplingProtocol: Sweeping; eventDate: 4/19-27/1939; **Record Level:** institutionCode: EFC

##### Distribution

AF: Saudi Arabia [as “South-western part”]. PA: Algeria, Armenia, Austria, Belgium, Bulgaria, China, Croatia, Czech, Egypt, France, Germany, Greece, Hungary, Iran, Italy, Kyrgyzstan, Lithuania, Mongolia, Morocco, Netherlands, Poland, Romania, Russia, Serbia, Slovakia, Spain, Switzerland, Turkey, Turkmenistan, Ukraine.

**Figure 6.**
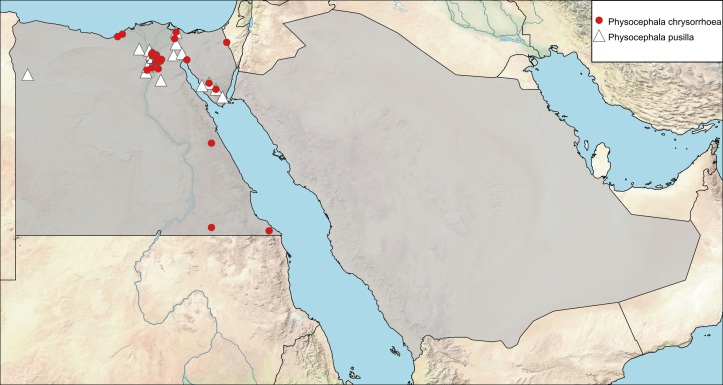
Local distribution map of *Physocephala chrysorrhoea* (Meigen) and *Physocephala pusilla* (Meigen) in Egypt and Saudi Arabia.

**Local distribution and dates of collection** (Fig. 6): EGYPT: Coastal Strip: Burg El-Arab, Mariout (February to May); Eastern Desert: Bir El-Fahm, Ogret El-Sheikh, Wadi El-Mallah, Wadi El-Maskhara, Wadi Garawi, Wadi Hoff, Wadi Morrah, Wadi Um-Elek, Wadi Zohleiga (March and April); Fayoum: Girza, Sennouris (January to March); Gebel Elba: locality unknown (January); Lower Nile Valley and Delta: Abu-Rawash, Barrage, Ezbet El-Nakhl, Hawamdiya, Helwan, Kafr Hakim, Kubba, Maadi, Mansouriah, Mazghouna (February to August); Sinai: Ein Gedeirat, Ein Moussa, Wadi El-Arbaein, Wadi El-Rabba (February to May); Western Desert: Dahshour (April) [Sources: Azmy (2016) and museum material in EFC and ESEC]. SAUDI ARABIA: locality and date unknown [Sources: Stuke (2017)].

#### Physocephala pusilla

(Meigen, 1804)

6CEC5DCC-417E-50CD-A2FF-8D365781D445

https://www.gbif.org/species/1568897

Conops pusilla Meigen, 1824: 280. Type locality: Germany (Frankreich).
Conops lacera Meigen, 1824: 130. Type locality: Austria.
Conops pumila Macquart, 1835: 26. Type locality: France.
Conops persica Becker, 1913: 609. Type locality: Iran.
Conops punctithorax Becker, 1913: 611. Type locality: Iran.

##### Materials

- **Type status:** Other material. **Occurrence:** recordedBy: R.M.; sex: 1 male; lifeStage: adult; **Taxon:** taxonID: https://www.gbif.org/species/1568897; scientificName: Physocephala pusilla; kingdom: Animalia; phylum: Arthropoda; class: Insecta; order: Diptera; family: Conopidae; **Location:** country: Egypt; locality: Abu-Rawash; decimalLatitude: 30.043845; decimalLongitude: 31.092932; **Identification:** identifiedBy: M. El-Hawagry; dateIdentified: June 2020; **Event:** samplingProtocol: Sweeping; eventDate: 05-26-1926; **Record Level:** institutionCode: EFC
- **Type status:** Other material. **Occurrence:** recordedBy: R.M.; sex: 1 male; lifeStage: adult; **Taxon:** taxonID: https://www.gbif.org/species/1568897; scientificName: Physocephala pusilla; kingdom: Animalia; phylum: Arthropoda; class: Insecta; order: Diptera; family: Conopidae; **Location:** country: Egypt; locality: Abu-Rawash; decimalLatitude: 30.043845; decimalLongitude: 31.092932; **Identification:** identifiedBy: M. El-Hawagry; dateIdentified: June 2020; **Event:** samplingProtocol: Sweeping; eventDate: 07-18-1925; **Record Level:** institutionCode: EFC
- **Type status:** Other material. **Occurrence:** recordedBy: Bdair; sex: 1 female; lifeStage: adult; **Taxon:** taxonID: https://www.gbif.org/species/1568897; scientificName: Physocephala pusilla; kingdom: Animalia; phylum: Arthropoda; class: Insecta; order: Diptera; family: Conopidae; **Location:** country: Egypt; locality: Ezbet-Naghl; decimalLatitude: 32.1111; decimalLongitude: 32.1625; **Identification:** identifiedBy: M. El-Hawagry; dateIdentified: June 2020; **Event:** samplingProtocol: Sweeping; eventDate: 05-06-1916; **Record Level:** institutionCode: EFC
- **Type status:** Other material. **Occurrence:** recordedBy: R.M.; sex: 1 male; lifeStage: adult; **Taxon:** taxonID: https://www.gbif.org/species/1568897; scientificName: Physocephala pusilla; kingdom: Animalia; phylum: Arthropoda; class: Insecta; order: Diptera; family: Conopidae; **Location:** country: Egypt; locality: Kafr Hakim; decimalLatitude: 30.0808; decimalLongitude: 31.1164; **Identification:** identifiedBy: M. El-Hawagry; dateIdentified: June 2020; **Event:** samplingProtocol: Sweeping; eventDate: 05-20-1930; **Record Level:** institutionCode: EFC

##### Distribution

PA: Afghanistan, China, Egypt, Europe (widespread), Kazakhstan, Mongolia, Morocco, Saudi Arabia, Syria, Tunisia, Turkmenistan.

**Local distribution and dates of collection** (Fig. 6): EGYPT: Eastern Desert: Cairo-Suez Road, El-Wasfia, Geneifa, Wadi Digla, Wadi Gharagid (April to September); Fayoum: Fayoum City, Kom Osheem (March to October); Lower Nile Valley and Delta: Abu-Rawash, Ashmoun, Ezbet El-Nakhl, Kafr Hakim, Maadi, Mansouriah, Pyramids (April to October); Sinai: Wadi El-Lega, Wadi El-Rabba, Wadi Firan, Wadi Isla, Wadi Khoshbi (April to August); Western Desert: Madinet El-Sadat, Siwa Oasis (July to October) [Sources: Zalat et al. (2008), Azmy (2016) and museum material in EFC and ESEC]. SAUDI ARABIA: locality and date unknown [Source: Abu-Zoherah et al. (1993) as *Conops lacera*].

#### Physocephala variegata

(Meigen, 1824)

B5A64EBE-FF12-52AE-A76D-CE6400E12594

https://www.gbif.org/species/1569101

Conops variegata Meigen, 1824: 132. Type locality: Austria & France.

##### Materials

- **Type status:** Other material. **Occurrence:** recordedBy: H.C.E & M.T.; sex: 1 male; lifeStage: adult; **Taxon:** taxonID: https://www.gbif.org/species/1569101; scientificName: Physocephala variegata; kingdom: Animalia; phylum: Arthropoda; class: Insecta; order: Diptera; family: Conopidae; **Location:** country: Egypt; locality: Dar El-Maskhara; decimalLatitude: 29.7833; decimalLongitude: 31.4167; **Identification:** identifiedBy: M. El-Hawagry; dateIdentified: June 2020; **Event:** samplingProtocol: Sweeping; eventDate: 04-29-1925; **Record Level:** institutionCode: EFC
- **Type status:** Other material. **Occurrence:** recordedBy: Efflatoun; sex: 1 male; lifeStage: adult; **Taxon:** taxonID: https://www.gbif.org/species/1569101; scientificName: Physocephala variegata; kingdom: Animalia; phylum: Arthropoda; class: Insecta; order: Diptera; family: Conopidae; **Location:** country: Egypt; locality: Wadi Digla; decimalLatitude: 29.9578; decimalLongitude: 31.3348; **Identification:** identifiedBy: M. El-Hawagry; dateIdentified: June 2020; **Event:** samplingProtocol: Sweeping; eventDate: 07-25-1926; **Record Level:** institutionCode: EFC
- **Type status:** Other material. **Occurrence:** recordedBy: Kassem; sex: 1 male; lifeStage: adult; **Taxon:** taxonID: https://www.gbif.org/species/1569101; scientificName: Physocephala variegata; kingdom: Animalia; phylum: Arthropoda; class: Insecta; order: Diptera; family: Conopidae; **Location:** country: Egypt; locality: Wadi Gharagid; decimalLatitude: 28.9550; decimalLongitude: 31.4822; **Identification:** identifiedBy: M. El-Hawagry; dateIdentified: June 2020; **Event:** samplingProtocol: Sweeping; eventDate: 08-24-1925; **Record Level:** institutionCode: EFC
- **Type status:** Other material. **Occurrence:** recordedBy: Farag; sex: 1 female; lifeStage: adult; **Taxon:** taxonID: https://www.gbif.org/species/1569101; scientificName: Physocephala variegata; kingdom: Animalia; phylum: Arthropoda; class: Insecta; order: Diptera; family: Conopidae; **Location:** country: Egypt; locality: Wadi Hoff; decimalLatitude: 29.8820558; decimalLongitude: 31.3109855; **Identification:** identifiedBy: M. El-Hawagry; dateIdentified: June 2020; **Event:** samplingProtocol: Sweeping; eventDate: 05-17-1928; **Record Level:** institutionCode: EFC
- **Type status:** Other material. **Occurrence:** recordedBy: Tewfik; sex: 1 female; lifeStage: adult; **Taxon:** taxonID: https://www.gbif.org/species/1569101; scientificName: Physocephala variegata; kingdom: Animalia; phylum: Arthropoda; class: Insecta; order: Diptera; family: Conopidae; **Location:** country: Egypt; locality: Wadi Hoff; decimalLatitude: 29.8820558; decimalLongitude: 31.3109855; **Identification:** identifiedBy: M. El-Hawagry; dateIdentified: June 2020; **Event:** samplingProtocol: Sweeping; eventDate: 06-06-1927; **Record Level:** institutionCode: EFC
- **Type status:** Other material. **Occurrence:** recordedBy: Efflatoun; sex: 1 female; lifeStage: adult; **Taxon:** taxonID: https://www.gbif.org/species/1569101; scientificName: Physocephala variegata; kingdom: Animalia; phylum: Arthropoda; class: Insecta; order: Diptera; family: Conopidae; **Location:** country: Egypt; locality: Halaib; decimalLatitude: 22.2203; decimalLongitude: 36.6427; **Identification:** identifiedBy: M. El-Hawagry; dateIdentified: June 2020; **Event:** samplingProtocol: Sweeping; eventDate: 01-12-1929; **Record Level:** institutionCode: EFC
- **Type status:** Other material. **Occurrence:** sex: 1 male; lifeStage: adult; **Taxon:** taxonID: https://www.gbif.org/species/1569101; scientificName: Physocephala variegata; kingdom: Animalia; phylum: Arthropoda; class: Insecta; order: Diptera; family: Conopidae; **Location:** country: Egypt; locality: W. Ibib; decimalLatitude: 22.83333; decimalLongitude: 35.76667; **Identification:** identifiedBy: M. El-Hawagry; dateIdentified: June 2020; **Event:** samplingProtocol: Sweeping; eventDate: 03-11-1928; **Record Level:** institutionCode: EFC
- **Type status:** Other material. **Occurrence:** recordedBy: R.M.; sex: 1 male; lifeStage: adult; **Taxon:** taxonID: https://www.gbif.org/species/1569101; scientificName: Physocephala variegata; kingdom: Animalia; phylum: Arthropoda; class: Insecta; order: Diptera; family: Conopidae; **Location:** country: Egypt; locality: Kafr Katati; decimalLatitude: 30.004146; decimalLongitude: 31.121566; **Identification:** identifiedBy: M. El-Hawagry; dateIdentified: June 2020; **Event:** samplingProtocol: Sweeping; eventDate: 05-25-1927; **Record Level:** institutionCode: EFC
- **Type status:** Other material. **Occurrence:** recordedBy: Sh.M.; sex: 1 male, 1 female; lifeStage: adult; **Taxon:** taxonID: https://www.gbif.org/species/1569101; scientificName: Physocephala variegata; kingdom: Animalia; phylum: Arthropoda; class: Insecta; order: Diptera; family: Conopidae; **Location:** country: Egypt; locality: Ein Gedeirat; decimalLatitude: 30.6500; decimalLongitude: 34.4333; **Identification:** identifiedBy: M. El-Hawagry; dateIdentified: June 2020; **Event:** samplingProtocol: Sweeping; eventDate: 06-25-1934; **Record Level:** institutionCode: EFC

##### Distribution

AF: Egypt [as “Gebel Elba”]. PA: Austria, Bulgaria, Egypt, France, Greece, Hungary, Iran, Kazakhstan, Kyrgyzstan, Mongolia, Romania, Russia, Saudi Arabia, Serbia, Turkey, Turkmenistan, Ukraine.

**Figure 7.**
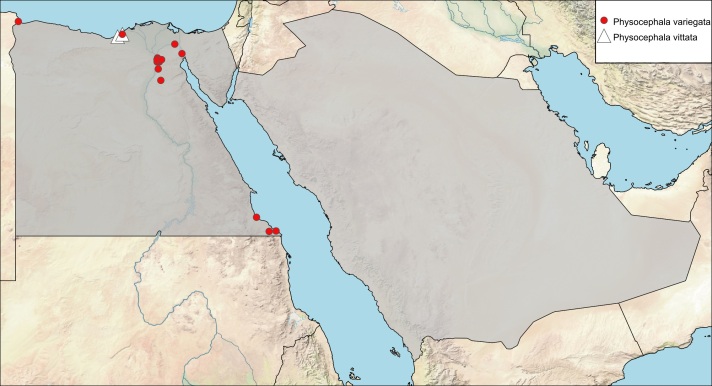
Local distribution map of *Physocephala variegata* (Meigen) and *Physocephala vittata* (Fabricius) in Egypt and Saudi Arabia.

**Local distribution and dates of collection** (Fig. 7): EGYPT: Coastal Strip: Mariout, Salloum (May to August); Eastern Desert: Abu Sueir, Geneifa, Wadi Digla, Wadi Dir El-Maskhara, Wadi Garrariyat, Wadi Gharagid, Wadi Hoff, Wadi Ibtadi, Wadi Rishrash, Wadi Um-Elek (April to August); Gebel Elba: Mersa Halaib, Wadi Edeib, Wadi Ibib (January to March); Lower Nile Valley and Delta: Abu-Rawash, Kafr Ghatati (May to July); Sinai: El-Arish, Wadi El-Daiqa, Wadi El-Rabba, Wadi Firan, Wadi Gedeirat (April to August) [Sources: Kröber (1927), Kröber (1929), Azmy (2016) and museum material in EFC and ESEC]. SAUDI ARABIA: locality and date unknown [Source: Zimina (1963), Stuke (2017)].

#### Physocephala vittata

(Fabricius, 1794)

86C49695-1BE7-59CB-8642-60F1BC8B84A8

https://www.gbif.org/species/1569046

Conops vittata Fabricius, 1794: 392. Type locality: Germany (Kiel).
Conops dorsalis Wiedemann in Meigen, 1824: 133. Type locality: Austria.
Conops solaeformis Gimmerthal, 1842: 672. Type locality: Latvia.
Conops semiatrata Costa, 1844: 89. Type locality: Italy.
Conops fraternus Loew, 1847: 18. Type locality: Turkey, Greece, Croatia & Italy.
Conops truncata Loew, 1847: 21. Type locality: Italy (Sicily).
Physocephala detecta Becker, 1913: 615. Type locality: Iran.
Physocephala vittata var. abdominalis Kröber, 1915: 57. Type locality: Turkey, Syria & Cyprus.
Physocephala vittata var. semirufa Kröber, 1915: 58. Type locality: Israel.
Physocephala truncata var. maculigera Kröber, 1915: 71. Type locality: Egypt, Tunisia, Algeria & Syria.
Physocephala vittata var. immaculata Kröber, 1939: 365. Type locality: Cyprus.

##### Distribution

AF: Ethiopia, Kenya, Yemen. PA: Afghanistan, Algeria, Armenia, Austria, Belarus, Belgium, Bulgaria, China, Croatia, Cyprus, Czech Republic, Egypt, France, Germany, Hungary, Iran, Israel, Italy, Lithuania, Malta, Mongolia, Morocco, Netherlands, Norway, Palestinian Territories, Poland, Portugal, Romania, Russia, Serbia, Slovakia, Spain, Sweden, Switzerland, Syria, Tunisia, Turkey, Turkmenistan, Ukraine, Uzbekistan.

**Local distribution and dates of collection** (Fig. 7): EGYPT: Coastal Strip: El-Burg, Mariout (May); Sinai: locality and date unknown; Upper Nile Valley: locality and date unknown [Sources: Kröber (1915a), Kröber (1927), Azmy (2016)].

#### MYOPINAE

CBC63EAC-B7F2-5B01-A625-1D7784F019FA

#### MYOPINI

113B4D60-A103-5B85-8BE8-F621A2CB8191

#### Myopa

Fabricius

A1376120-EB6D-5604-AD52-58567D1CB98E

https://www.gbif.org/species/1568357

Myopa Fabricius, 1775: 798. Type species: *Conops buccata* Linnaeus, by subsequent designation of Curtis (1831).

#### Myopa dorsalis

Fabricius, 1794

4561A5BB-F557-5A95-A89B-4FC0F211ACC5

https://www.gbif.org/species/1568413

Myopa dorsalis Fabricius, 1794: 397. Type locality: Germany.
Myopa ferruginea Panzer, 1794: 24. Type locality: Germany.
Myopa grandis Meigen, 1804: 284. Type locality: Germany.
Myopa dorsalis var. nigrifacies Becker, 1922: 289. Type locality: Not given.

##### Materials

- **Type status:** Other material. **Occurrence:** recordedBy: Tewfik; sex: 1 male; lifeStage: adult; **Taxon:** taxonID: https://www.gbif.org/species/1568413; scientificName: Myopa dorsalis; kingdom: Animalia; phylum: Arthropoda; class: Insecta; order: Diptera; family: Conopidae; **Location:** country: Egypt; locality: Behig; decimalLatitude: 30.9310; decimalLongitude: 29.5988; **Identification:** identifiedBy: M. El-Hawagry; dateIdentified: June 2020; **Event:** samplingProtocol: Sweeping; eventDate: 02-28-1927; **Record Level:** institutionCode: EFC
- **Type status:** Other material. **Occurrence:** recordedBy: Tewfik; sex: 1 male; lifeStage: adult; **Taxon:** taxonID: https://www.gbif.org/species/1568413; scientificName: Myopa dorsalis; kingdom: Animalia; phylum: Arthropoda; class: Insecta; order: Diptera; family: Conopidae; **Location:** country: Egypt; locality: Burg El-Arab; decimalLatitude: 30.908084; decimalLongitude: 29.546389; **Identification:** identifiedBy: M. El-Hawagry; dateIdentified: June 2020; **Event:** samplingProtocol: Sweeping; eventDate: 02-10-1926; **Record Level:** institutionCode: EFC

##### Distribution

OR: India. PA: Albania, Algeria, Armenia, Austria, Belarus, Belgium, Bulgaria, China, Croatia, Czech Republic, Denmark, Egypt, Finland, France, Germany, Greece, Hungary, Iran, Italy, Kazakhstan, Luxembourg, Morocco, Netherlands, Norway, Poland, Romania, Russia, Slovakia, Spain, Sweden, Switzerland, Syria, Tunisia, Turkey, Turkmenistan, Ukraine.

**Figure 8.**
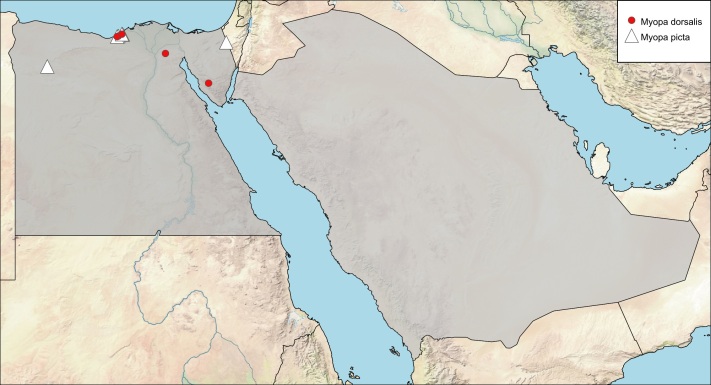
Local distribution map of *Myopa dorsalis* Fabricius and *Myopa picta* Panzer in Egypt and Saudi Arabia.

**Local distribution and dates of collection** (Fig. 8): EGYPT: Coastal Strip: Behig, Burg El-Arab, Mariout (February and March); Lower Nile Valley and Delta: Madinet Badr (January); Sinai: Wadi El-Rabba (April) [Sources: Azmy et al. (2016) and museum material in EFC and ESEC].

#### Myopa picta

Panzer, 1798

2657AEC4-4949-5D2C-8006-B3693AFC63B2

https://www.gbif.org/species/1568425

Myopa picta Panzer, 1798: 22. Type locality: Austria.
Myopa varia Wiedemann, 1830: 242. Type locality: Egypt.
Myopa meridionalis Macquart, 1835: 34. Type locality: Egypt.

##### Materials

- **Type status:** Other material. **Occurrence:** recordedBy: Tewfik; sex: 1 male, 1 female; lifeStage: adult; **Taxon:** taxonID: https://www.gbif.org/species/1568425; scientificName: Myopa picta; kingdom: Animalia; phylum: Arthropoda; class: Insecta; order: Diptera; family: Conopidae; **Location:** country: Egypt; locality: Behig; decimalLatitude: 30.9310; decimalLongitude: 29.5988; **Identification:** identifiedBy: M. El-Hawagry; dateIdentified: June 2020; **Event:** samplingProtocol: Sweeping; eventDate: 02-28-1927; **Record Level:** institutionCode: EFC
- **Type status:** Other material. **Occurrence:** recordedBy: H.C.E & M.T.; sex: 1 male; lifeStage: adult; **Taxon:** taxonID: https://www.gbif.org/species/1568425; scientificName: Myopa picta; kingdom: Animalia; phylum: Arthropoda; class: Insecta; order: Diptera; family: Conopidae; **Location:** country: Egypt; locality: Burg; decimalLatitude: 30.908084; decimalLongitude: 29.546389; **Identification:** identifiedBy: M. El-Hawagry; dateIdentified: June 2020; **Event:** samplingProtocol: Sweeping; eventDate: 02-25-1932; **Record Level:** institutionCode: EFC
- **Type status:** Other material. **Occurrence:** recordedBy: Tewfik; sex: 1 male, 1 female; lifeStage: adult; **Taxon:** taxonID: https://www.gbif.org/species/1568425; scientificName: Myopa picta; kingdom: Animalia; phylum: Arthropoda; class: Insecta; order: Diptera; family: Conopidae; **Location:** country: Egypt; locality: Burg El-Arab; decimalLatitude: 30.908084; decimalLongitude: 29.546389; **Identification:** identifiedBy: M. El-Hawagry; dateIdentified: June 2020; **Event:** samplingProtocol: Sweeping; eventDate: 02-10-1926; **Record Level:** institutionCode: EFC
- **Type status:** Other material. **Occurrence:** recordedBy: Willson; sex: 1 male; lifeStage: adult; **Taxon:** taxonID: https://www.gbif.org/species/1568425; scientificName: Myopa picta; kingdom: Animalia; phylum: Arthropoda; class: Insecta; order: Diptera; family: Conopidae; **Location:** country: Egypt; locality: Mersa Matrouh; decimalLatitude: 29.5696; decimalLongitude: 26.4194; **Identification:** identifiedBy: M. El-Hawagry; dateIdentified: June 2020; **Event:** samplingProtocol: Sweeping; eventDate: March.1920; **Record Level:** institutionCode: EFC

##### Distribution

AF: Ethiopia. OR: India, Pakistan. PA: Afghanistan, Austria, Belarus, Belgium, Bosnia and Herzegovina, Bulgaria, China, Croatia, Czech Republic, Egypt, France, Germany, Greece, Iran, Italy, Kyrgyzstan, Morocco, Poland, Romania, Russia, Slovakia, Spain, Switzerland, Tunisia, Turkey, Turkmenistan, Ukraine.

**Local distribution and dates of collection** (Fig. 8): EGYPT: Coastal Strip: Amria, Behig, Burg El-Arab, Mariout, Mersa Matrouh (February to May); Sinai: Ein Gedeirat (June) [Sources: Kröber (1925), Azmy et al. (2016) and museum material in EFC and ESEC].

#### Myopa stigma

Meigen, 1824

33C3A52E-EDDF-5238-8854-C8C13C857252

https://www.gbif.org/species/1568382

Myopa stigma Meigen, 1824: 148. Type locality: Austria.
Myopa arabica Macquart 1851: 138. Type locality: Saudi Arabia (Jeddah).

##### Distribution

AF: Ethiopia. PA: Afghanistan, Algeria, Austria, Belarus, Bulgaria, Cyprus, Czech Republic, France, Germany, Greece, Hungary, Italy, Kazakhstan, Morocco, Poland, Portugal, Romania, Russia, Saudi Arabia (Jeddah), Slovakia, Spain, Switzerland, Tunisia, Turkey, Ukraine.

**Figure 9.**
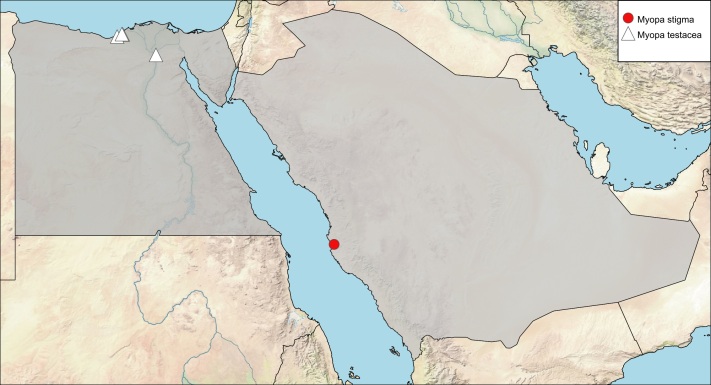
Local distribution map of *Myopa stigma* Meigen and *Myopa testacea* (Linnaeus) in Egypt and Saudi Arabia.

**Local distribution and dates of collection** (Fig. 9): SAUDI ARABIA: Makka Al-Mukarramah: Jeddah (date unknown) [Source: original description of *M. arabica* Macquart and Stuke and Clements 2008].

#### Myopa testacea

(Linnaeus, 1767)

5DBDE653-63AB-533E-8BF5-2E34BCA1A11D

https://www.gbif.org/species/1568398

Conops testacea Linnaeus, 1767: 1006. Type locality: "Europa australis Ascanius".
Myopa longirostris Robineau-Desvoidy, 1830: 243. Type locality: Not given.
Myopa pictipennis Robineau-Desvoidy, 1830: 243. Type locality: Not given.
Myopa umbripennis Robineau-Desvoidy, 1830: 243. Type locality: Not given.
Myopa testacea var. japonica Kröber, 1916: 89. Type locality: Japan.

##### Materials

- **Type status:** Other material. **Occurrence:** recordedBy: H.C.E & M.T.; sex: 1 male, 1 female; lifeStage: adult; **Taxon:** taxonID: https://www.gbif.org/species/1568398; scientificName: Myopa testacea; kingdom: Animalia; phylum: Arthropoda; class: Insecta; order: Diptera; family: Conopidae; **Location:** country: Egypt; locality: Burg; decimalLatitude: 30.9310; decimalLongitude: 29.5988; **Identification:** identifiedBy: M. El-Hawagry; dateIdentified: June 2020; **Event:** samplingProtocol: Sweeping; eventDate: 02-25-1926; **Record Level:** institutionCode: EFC
- **Type status:** Other material. **Occurrence:** recordedBy: Tewfik; sex: 1 female; lifeStage: adult; **Taxon:** taxonID: https://www.gbif.org/species/1568398; scientificName: Myopa testacea; kingdom: Animalia; phylum: Arthropoda; class: Insecta; order: Diptera; family: Conopidae; **Location:** country: Egypt; locality: Burg El-Arab; decimalLatitude: 30.908084; decimalLongitude: 29.546389; **Identification:** identifiedBy: M. El-Hawagry; dateIdentified: June 2020; **Event:** samplingProtocol: Sweeping; eventDate: 02-10-1926; **Record Level:** institutionCode: EFC
- **Type status:** Other material. **Occurrence:** recordedBy: H.C.E & M.T.; sex: 1 male; lifeStage: adult; **Taxon:** taxonID: https://www.gbif.org/species/1568398; scientificName: Myopa testacea; kingdom: Animalia; phylum: Arthropoda; class: Insecta; order: Diptera; family: Conopidae; **Location:** country: Egypt; locality: Kubba; decimalLatitude: 30.0876; decimalLongitude: 31.2854; **Identification:** identifiedBy: M. El-Hawagry; dateIdentified: June 2020; **Event:** samplingProtocol: Sweeping; eventDate: 02-20-1921; **Record Level:** institutionCode: ESEC

##### Distribution

OR: India. PA: Afghanistan, Austria, Belarus, Belgium, Bulgaria, China, Croatia, Czech Republic, Denmark, Egypt, Finland, France, Germany, Great Britain, Greece, Hungary, Ireland, Italy, Japan, Kazakhstan, Kyrgyzstan, Lithuania, Luxembourg, Malta, Mongolia, Netherlands, Norway, Poland, Portugal, Romania, Russia, Slovakia, Spain, Sweden, Switzerland, Tunisia, Turkey, Turkmenistan, Ukraine.

**Local distribution and dates of collection** (Fig. 9): EGYPT: Coastal Strip: Amria, Burg El-Arab, Mariout (February to June); Lower Nile Valley and Delta: Kubba (February) [Sources: Azmy et al. (2016) and museum material in EFC and ESEC].

#### THECOPHORINI

F28F8D07-2E94-5BAC-8C4A-460D355BB105

#### Thecophora

Rondani

4725D923-F9C0-5829-9C2C-7A4F875AC92F

Thecophora ***Thecophora*** Rondani, 1845: 15. Type species: *Myopa atra* Fabricius, by monotypy.

#### Thecophora atra

(Fabricius, 1775)

C9C45211-4E80-5505-9B32-38BB065160CD

https://www.gbif.org/species/1569366

Myopa atra Fabricius, 1775: 799. Type locality: Denmark (Copenhagen).
Myopa annulata Fabricius, 1794: 399. Type locality: Italy.
Myopa maculata Meigen, 1804: 287. Type locality: France.
Myopa micans Meigen, 1804: 288. Type locality: France.
Myopa femorata Fabricius, 1805: 181. Type locality: Germany.
Occemya fulvifrons Robineau-Desvoidy, 1853: 134. Type locality: France.
Occemya meigeni Robineau-Desvoidy, 1853: 135. Type locality: France.
Occemya grisea Robineau-Desvoidy, 1853: 137. Type locality: France.
Occemya lamarckii Robineau-Desvoidy, 1853: 140. Type locality: France.
Occemya guerini Robineau-Desvoidy, 1853: 141. Type locality: France.
Occemya bigoti Robineau-Desvoidy, 1853: 142. Type locality: France.
Occemya brunipes Robineau-Desvoidy, 1853: 143. Type locality: France.
Occemya lucasi Robineau-Desvoidy, 1853: 144. Type locality: France.

##### Materials

- **Type status:** Other material. **Occurrence:** recordedBy: Efflatoun; sex: 1 male; lifeStage: adult; **Taxon:** taxonID: https://www.gbif.org/species/1569366; scientificName: Thecophora atra; kingdom: Animalia; phylum: Arthropoda; class: Insecta; order: Diptera; family: Conopidae; **Location:** country: Egypt; locality: Ezbet El-Nakhl; decimalLatitude: 32.1111; decimalLongitude: 32.1625; **Identification:** identifiedBy: M. El-Hawagry; dateIdentified: June 2020; **Event:** samplingProtocol: Sweeping; eventDate: 04-25-1924; **Record Level:** institutionCode: EFC
- **Type status:** Other material. **Occurrence:** recordedBy: Efflatoun; sex: 1 female; lifeStage: adult; **Taxon:** taxonID: https://www.gbif.org/species/1569366; scientificName: Thecophora atra; kingdom: Animalia; phylum: Arthropoda; class: Insecta; order: Diptera; family: Conopidae; **Location:** country: Egypt; locality: Hawamdia; decimalLatitude: 29.9092; decimalLongitude: 31.2615; **Identification:** identifiedBy: M. El-Hawagry; dateIdentified: June 2020; **Event:** samplingProtocol: Sweeping; eventDate: August.1925; **Record Level:** institutionCode: EFC
- **Type status:** Other material. **Occurrence:** recordedBy: Farag; sex: 1 female; lifeStage: adult; **Taxon:** taxonID: https://www.gbif.org/species/1569366; scientificName: Thecophora atra; kingdom: Animalia; phylum: Arthropoda; class: Insecta; order: Diptera; family: Conopidae; **Location:** country: Egypt; locality: Helwan; decimalLatitude: 29.8500; decimalLongitude: 31.3333; **Identification:** identifiedBy: M. El-Hawagry; dateIdentified: June 2020; **Event:** samplingProtocol: Sweeping; eventDate: 12-11-1934; **Record Level:** institutionCode: EFC
- **Type status:** Other material. **Occurrence:** recordedBy: El-Hawagry; sex: 1 female; lifeStage: adult; **Taxon:** taxonID: https://www.gbif.org/species/1569366; scientificName: Thecophora atra; kingdom: Animalia; phylum: Arthropoda; class: Insecta; order: Diptera; family: Conopidae; **Location:** country: Saudi Arabia; locality: Al-Aqiq; **Identification:** identifiedBy: M. El-Hawagry; dateIdentified: June 2020; **Event:** samplingProtocol: Sweeping; eventDate: 4-20-2011; **Record Level:** institutionCode: MSHC

##### Distribution

AF: Saudi Arabia [as “South-western part”] (first record). OR: India, Sri Lanka. PA: Afghanistan, Albania, Algeria, Austria, Belgium, Bosnia and Herzegovina, Bulgaria, China, Croatia, Cyprus, Czech Republic, Denmark, Egypt, Finland, France, Germany, Great Britain, Greece, Hungary, Iran, Ireland, Italy, Japan, Kyrgyzstan, Lithuania, Luxembourg, Macedonia, Malta, Mongolia, Montenegro, Morocco, Netherlands, North Korea, Norway, Poland, Portugal, Romania, Russia, Serbia, Slovakia, Spain, Sweden, Switzerland, Tunisia, Turkey, Turkmenistan, Ukraine.

**Figure 10.**
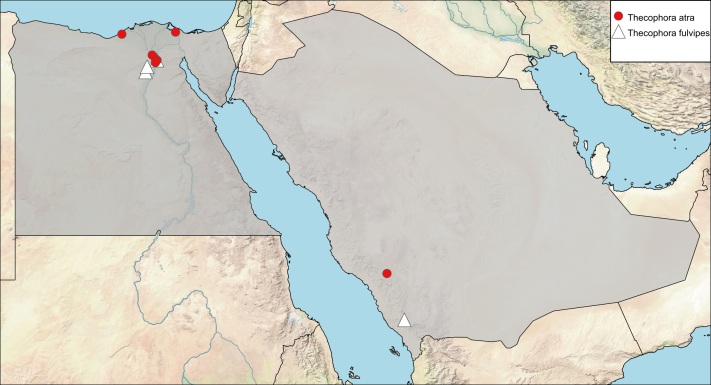
Local distribution map of *Thecophora atra* (Fabricius) and *Thecophora fulvipes* (Robineau-Desvoidy) in Egypt and Saudi Arabia.

**Local distribution and dates of collection** (Fig. 10): EGYPT: Coastal Strip: Mariout (April and May); Lower Nile Valley and Delta: Ezbet El-Nakhl, Hawamdia, Helwan, Kafr Hakim, Mazghouna (March to December) [Sources: Azmy et al. 2016 and museum material in EFC and ESEC]. SAUDI ARABIA: Al-Baha: Al-Aqiq (April) [Source: collected material].

##### Notes

This species (Fig. 12b) is recorded herein for the first time from Saudi Arabia and this is the first record in the Afrotropical Region considering the south-western part of Saudi Arabia to be affiliated with the Afrotropical Region.

#### Thecophora fulvipes

(Robineau-Desvoidy, 1830)

C580F758-DF0E-52A7-A4B8-24E460A2EEA4

https://www.gbif.org/species/1569429

Myopa fulvipes Robineau-Desvoidy, 1830: 246. Type locality: France (Paris).
Myopa sundewalli Zetterstedt, 1844: 942. Type locality: Sweden.

##### Materials

- **Type status:** Other material. **Occurrence:** recordedBy: Aldhafer H.M. et al.; sex: 1 female; lifeStage: adult; **Taxon:** taxonID: https://www.gbif.org/species/1569429; scientificName: Thecophora fulvipes; kingdom: Animalia; phylum: Arthropoda; class: Insecta; order: Diptera; family: Conopidae; **Location:** country: Saudi Arabia; stateProvince: Asir; locality: Garf Raydah Nature Reserve; decimalLatitude: 18.194917; decimalLongitude: 42.4072; **Identification:** identifiedBy: M. El-Hawagry; dateIdentified: June 2020; **Event:** samplingProtocol: Malaise trap; eventDate: 06-07-2014; **Record Level:** institutionCode: KSMA

##### Distribution

AF: Saudi Arabia [as “South-western part”] (El-Hawagry et al. 2017El-Hawagry et al. 2017). PA: Austria, Belgium, Bulgaria, China, Croatia, Czech Republic, Denmark, Estonia, Finland, France, Germany, Great Britain, Hungary, Iran, Ireland, Italy, Latvia, Lithuania, Mongolia, Netherlands, Poland, Portugal, Romania, Russia, Serbia, Slovakia, Spain, Sweden, Switzerland, Turkey, Ukraine.

**Local distribution and dates of collection** (Fig. 10): EGYPT: Fayoum: Kom Osheem (June); Lower Nile Valley and Delta: Helwan (December) [Sources: Azmy 2016]. SAUDI ARABIA: Asir: Raydah Nature Reserve (June) [Source: El-Hawagry et al. 2017 and collected material].

#### ZODIONINAE

31F6A9C8-4794-58AF-90F5-A2645E6B4DED

#### Zodion

Latreille

7C6AD3BD-43E6-5BB2-BC92-2A7FB81957C9

https://www.gbif.org/species/1568754

Zodion ***Zodion*** Latreille, 1797: 162. Type species: *Myopa cinerea* Fabricius, by Subsequent monotypy of Latreille (1802).

#### Zodion cinereum

(Fabricius, 1794)

B669E2C8-1C91-559F-A10D-8DB9A0F9AE59

https://www.gbif.org/species/1568846

Myopa cinerea Fabricius, 1794: 399. Type locality: Italy.
Myopa notata Meigen, 1804: 288. Type locality: France.
Myopa tibialis Fabricius, 1805: 182. Type locality: Europe.
Zodion conopsoides Latreille, 1809: 337. Type locality: France.
Zodion pedicillatum Robineau-Desvoidy, 1830: 252. Type locality: Not given.
Zodion fuliginosum Robineau-Desvoidy, 1853: 156. Type locality: France.
Zodion fulvipes Robineau-Desvoidy, 1853: 157. Type locality: France.
Zodion fulvicorne Robineau-Desvoidy, 1853: 158. Type locality: France.
Zodion cinereum var. rubescens Szilády, 1925: 223. Type locality: Hungary.
Zodion cinereum var. kerteszi Szilády, 1926: 589. Type locality: Hungary.

##### Materials

- **Type status:** Other material. **Occurrence:** recordedBy: Tewfik; sex: 1 male, 1 female; lifeStage: adult; **Taxon:** taxonID: https://www.gbif.org/species/1568846; scientificName: Zodion cinereum; kingdom: Animalia; phylum: Arthropoda; class: Insecta; order: Diptera; family: Conopidae; **Location:** country: Egypt; locality: Behig; decimalLatitude: 30.9310; decimalLongitude: 29.5988; **Identification:** identifiedBy: M. El-Hawagry; dateIdentified: June 2020; **Event:** samplingProtocol: Sweeping; eventDate: 02-28-1927; **Record Level:** institutionCode: EFC
- **Type status:** Other material. **Occurrence:** recordedBy: H.C.E & M.T.; sex: 1 male, 1 female; lifeStage: adult; **Taxon:** taxonID: https://www.gbif.org/species/1568846; scientificName: Zodion cinereum; kingdom: Animalia; phylum: Arthropoda; class: Insecta; order: Diptera; family: Conopidae; **Location:** country: Egypt; locality: Burg; decimalLatitude: 30.908084; decimalLongitude: 29.546389; **Identification:** identifiedBy: M. El-Hawagry; dateIdentified: June 2020; **Event:** samplingProtocol: Sweeping; eventDate: 03-05-1930; **Record Level:** institutionCode: EFC
- **Type status:** Other material. **Occurrence:** recordedBy: H.C.E & M.T.; sex: 1 male; lifeStage: adult; **Taxon:** taxonID: https://www.gbif.org/species/1568846; scientificName: Zodion cinereum; kingdom: Animalia; phylum: Arthropoda; class: Insecta; order: Diptera; family: Conopidae; **Location:** country: Egypt; locality: Burg; decimalLatitude: 30.908084; decimalLongitude: 29.546389; **Identification:** identifiedBy: M. El-Hawagry; dateIdentified: June 2020; **Event:** samplingProtocol: Sweeping; eventDate: 02-25-1932; **Record Level:** institutionCode: EFC
- **Type status:** Other material. **Occurrence:** recordedBy: Efflatoun; sex: 1 male; lifeStage: adult; **Taxon:** taxonID: https://www.gbif.org/species/1568846; scientificName: Zodion cinereum; kingdom: Animalia; phylum: Arthropoda; class: Insecta; order: Diptera; family: Conopidae; **Location:** country: Egypt; locality: Mariout; decimalLatitude: 31.017186; decimalLongitude: 29.759966; **Identification:** identifiedBy: M. El-Hawagry; dateIdentified: June 2020; **Event:** samplingProtocol: Sweeping; eventDate: 02-15-1923; **Record Level:** institutionCode: EFC
- **Type status:** Other material. **Occurrence:** recordedBy: H.C.E & M.T.; sex: 1 male; lifeStage: adult; **Taxon:** taxonID: https://www.gbif.org/species/1568846; scientificName: Zodion cinereum; kingdom: Animalia; phylum: Arthropoda; class: Insecta; order: Diptera; family: Conopidae; **Location:** country: Egypt; locality: Mariout: El-Burg; decimalLatitude: 30.908084; decimalLongitude: 29.546389; **Identification:** identifiedBy: M. El-Hawagry; dateIdentified: June 2020; **Event:** samplingProtocol: Sweeping; eventDate: 06-16-1929; **Record Level:** institutionCode: EFC
- **Type status:** Other material. **Occurrence:** recordedBy: El-Hawagry; sex: 1 male, 1 female; lifeStage: adult; **Taxon:** taxonID: https://www.gbif.org/species/1568846; scientificName: Zodion cinereum; kingdom: Animalia; phylum: Arthropoda; class: Insecta; order: Diptera; family: Conopidae; **Location:** country: Saudi Arabia; stateProvince: Al-Baha; locality: Ghabet Shahba; decimalLatitude: 20.02723; decimalLongitude: 41.28565; **Identification:** identifiedBy: M. El-Hawagry; dateIdentified: June 2020; **Event:** samplingProtocol: Sweeping; eventDate: 05-06-2012; **Record Level:** institutionCode: MSHC
- **Type status:** Other material. **Occurrence:** recordedBy: Aldhafer H.M. et al.; sex: 1 female; lifeStage: adult; **Taxon:** taxonID: https://www.gbif.org/species/1568846; scientificName: Zodion cinereum; kingdom: Animalia; phylum: Arthropoda; class: Insecta; order: Diptera; family: Conopidae; **Location:** country: Saudi Arabia; stateProvince: Al-Baha; locality: Jabal Shada al-A’la Nature Reserve; decimalLatitude: 19.8388; decimalLongitude: 41.3101; **Identification:** identifiedBy: M. El-Hawagry; dateIdentified: June 2020; **Event:** samplingProtocol: Malaise trap; eventDate: 05-05-2015; **Record Level:** institutionCode: KSMA

##### Distribution

AF: Central African Republic, Saudi Arabia [as “South-western part”] (El-Hawagry et al. 2016, United Arabian Emirates. OR: India. PA: Afghanistan, Albania, Algeria, Armenia, Austria, Belgium, Bosnia and Herzegovina, Bulgaria, China, Croatia, Cyprus, Czech Republic, Denmark, Egypt, Estonia, Finland, France, Germany, Great Britain, Greece, Hungary, Iran, Ireland, Italy, Japan, Kazakhstan, Kyrgyzstan, Latvia, Lebanon, Lithuania, Luxembourg, Macedonia, Moldova, Mongolia, Montenegro, Morocco, Netherlands, Norway, Poland, Portugal, Romania, Russia, Serbia, Slovakia, Spain, Sweden, Switzerland, Tunisia, Turkey, Turkmenistan, Ukraine.

**Figure 11.**
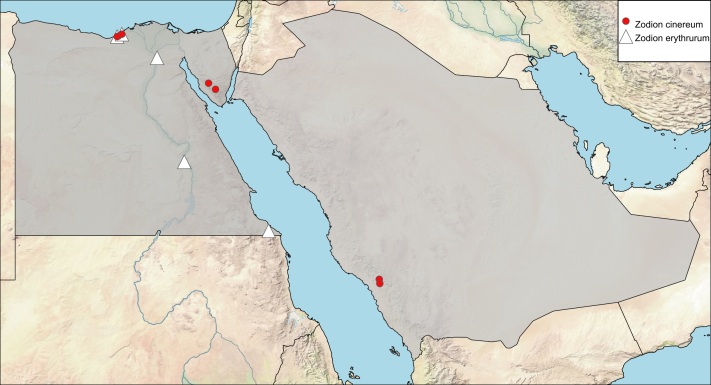
Local distribution map of *Zodion cinereum* (Fabricius) and *Zodion erythrurum* Rondani in Egypt and Saudi Arabia.

**Local distribution and dates of collection** (Fig. 11): EGYPT: Coastal Strip: Behig, Burg El-Arab, Mariout (February and March); Sinai: Wadi El-Lega, Wadi El-Rabba (April, August and September) [Sources: Azmy (2016) and museum material in EFC]. SAUDI ARABIA: Al-Baha: Ghabet Shahba (Al-Baha City) (May and June 2012), Jabal Shada al-A’la Nature Reserve (May) [Source: Stuke (2008), El-Hawagry et al. (2016) and collected material].

#### Zodion erythrurum

Rondani, 1865

A862FD02-D012-50A3-8142-283DF648131F

https://www.gbif.org/species/1568759

Zodion erythrurum Rondani, 1865: 146. Type locality: Italy (Etruria).
Zodion pulchrum Loew, 1868: 384. Type locality: Italy.
Zodion vittipes Strobl, 1906: 331. Type locality: Spain.

##### Distribution

PA: Algeria, Bulgaria, Egypt, France, Hungary, Israel, Italy, Kazakhstan, Kyrgyzstan, Morocco, Poland, Romania, Russia, Serbia, Spain, Tunisia, Turkey, Turkmenistan, Ukraine.

**Local distribution and dates of collection** (Fig. 11): EGYPT: Coastal Strip: Burg El-Arab, Mariout (February to April); Eastern Desert: Wadi Digla (July and August); Gebel Elba: locality and unknown (April and May); Upper Nile Valley: Esna (January) [Sources: Kröber (1927), Azmy (2016) and museum material in ESEC].

## Supplementary Material

XML Treatment for CONOPIDAE

XML Treatment for CONOPINAE

XML Treatment for CONOPINI

XML Treatment for Conops

XML Treatment for Asiconops

XML Treatment for Conops (Asiconops) elegans

XML Treatment for Conops (Asiconops) flavifrons

XML Treatment for Conops (Asiconops) nubeculipennis

XML Treatment for Conops

XML Treatment for Conops (Conops) flavicauda

XML Treatment for Conops (Conops) quadrifasciatus

XML Treatment for Conops (Conops) rufiventris

XML Treatment for Conops (Conops) tomentosus

XML Treatment for PHYSOCEPHALINI

XML Treatment for Physocephala

XML Treatment for Physocephala antiqua

XML Treatment for Physocephala chrysorrhoea

XML Treatment for Physocephala pusilla

XML Treatment for Physocephala variegata

XML Treatment for Physocephala vittata

XML Treatment for MYOPINAE

XML Treatment for MYOPINI

XML Treatment for Myopa

XML Treatment for Myopa dorsalis

XML Treatment for Myopa picta

XML Treatment for Myopa stigma

XML Treatment for Myopa testacea

XML Treatment for THECOPHORINI

XML Treatment for Thecophora

XML Treatment for Thecophora atra

XML Treatment for Thecophora fulvipes

XML Treatment for ZODIONINAE

XML Treatment for Zodion

XML Treatment for Zodion cinereum

XML Treatment for Zodion erythrurum

## Figures and Tables

**Figure 12a.**
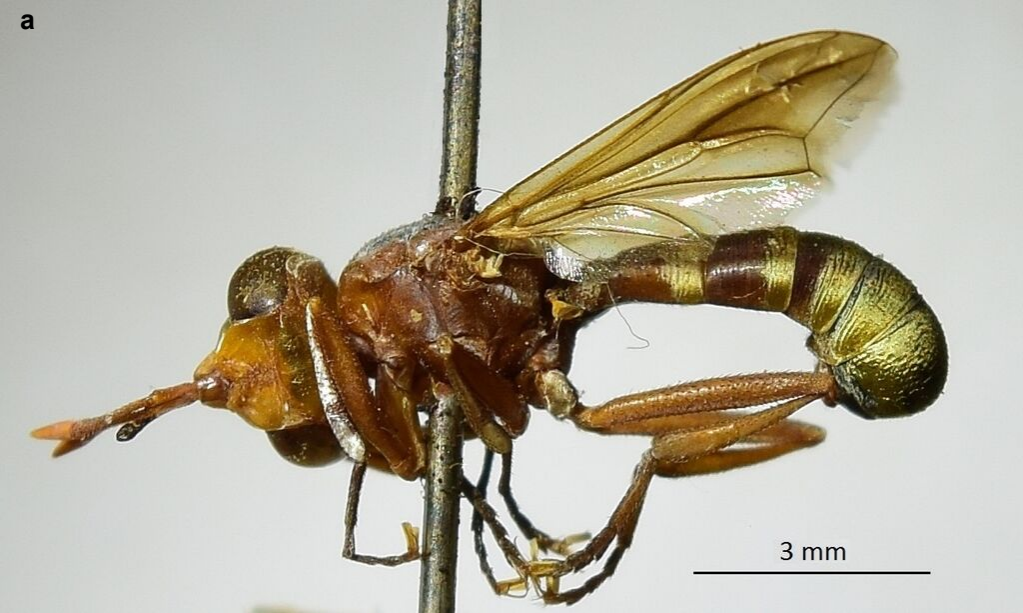
Conops (Asiconops) elegans Meigen male, lateral

**Figure 12b.**
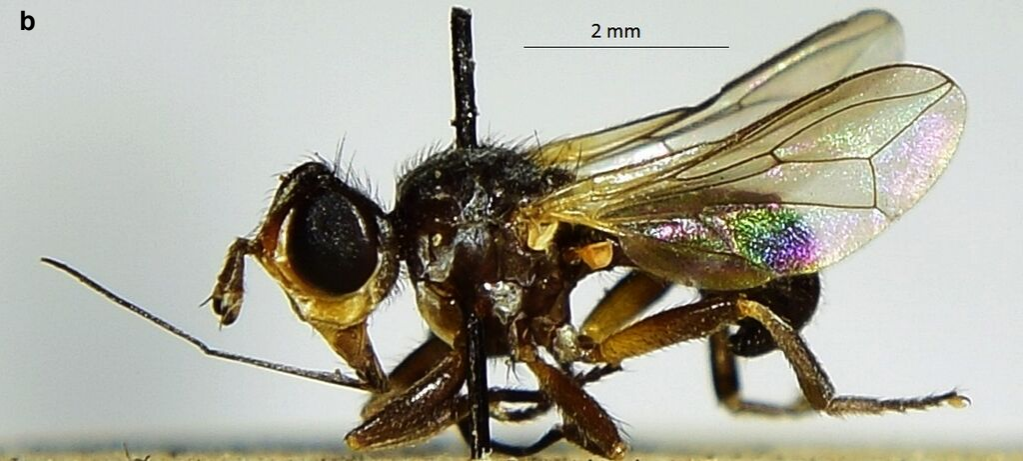
*Thecophora atra* (Fabricius) female, lateral

**Table 1.** Conopid species treated in the present study as recorded from Egypt and Saudi Arabia (* = recorded, - = not recorded).

**Species**	**Egypt**	**Saudi Arabia**
Subfamily CONOPINAE		
Tribe CONOPINI		
Conops (Asiconops) elegans Meigen, 1804	*	* (new record)
Conops (Asiconops) flavifrons Meigen, 1804	*	-
Conops (Asiconops) nubeculipennis Bezzi, 1901	*	*
Conops (Conops) flavicauda (Bigot, 1880)	*	-
Conops (Conops) quadrifasciatus De Geer, 1776	-	* (new record)
Conops (Conops) rufiventris Macquart, 1849	*	-
Conops (Conops) tomentosus Kröber, 1916	-	*
Tribe PHYSOCEPHALINI		
*Physocephala antiqua* (Wiedemann, 1830)	*	*
*Physocephala chrysorrhoea* (Meigen, 1824)	*	*
*Physocephala pusilla* (Meigen, 1804)	*	*
*Physocephala variegata* (Meigen, 1824)	*	*
*Physocephala vittata* (Fabricius, 1794)	*	-
Subfamily MYOPINAE		
Tribe MYOPINI		
*Myopa dorsalis* Fabricius, 1794	*	-
*Myopa picta* Panzer, 1798	*	-
*Myopa stigma* Meigen, 1824	-	*
*Myopa testacea* (Linnaeus, 1767)	*	-
Tribe THECOPHORINI		
*Thecophora atra* (Fabricius, 1775)	*	* (new record)
*Thecophora fulvipes* (Robineau-Desvoidy, 1830)	*	*
Subfamily ZODIONINAE		
*Zodion cinereum* (Fabricius, 1794)	*	*
*Zodion erythrurum* Rondani, 1865	*	-

**Table 2.** A gazetteer of some Egyptian and Saudi Arabian localities of the family Conopidae.

**Country**	**Locality**	**Governorate**	**Ecological zone or Region**	**Latitude (N) / Longitude (E)**
Egypt	Abu-Kir	Alexandria	Coastal Strip	31.22429, 29.94664
	Abu-Rawash	Giza	Lower Nile Valley and Delta	30.0438, 31.0929
	Abu-Sueir	Ismailia	Eastern Desert	30.5766, 32.1076
	Amria	Alexandria	Coastal Strip	31.0037, 29.7983
	Ashmoun	Menofiya	Lower Nile Valley and Delta	30.29414, 30.96941
	Aswan, south	Aswan	Upper Nile Valley	22.6651, 31.7943
	Balteem	Kafr Al-Sheikh	Coastal Strip	31.53811, 31.19606
	Barrage	Qalyoubia	Lower Nile Valley and Delta	30.1835, 31.1107
	Behig	Alexandria	Coastal Strip	30.931, 29.5988
	Burg	Alexandria	Coastal Strip	30.9081, 29.5464
	Burgash	Giza	Lower Nile Valley and Delta	30.1636, 31.0379
	Cairo	Cairo	Lower Nile Valley and Delta	30.0861, 31.2856
	Cairo-Suez Road	Cairo	Eastern Desert	30.0849, 32.0542
	Dahshour	Giza	Lower Nile Valley and Delta	29.7539, 31.2431
	Dakhla	New Valley	Western Desert	25.5, 29.1667
	Dekhela	Alexandria	Coastal Strip	31.12098, 29.81563
	Edfina	Al-Behaira	Lower Nile Valley and Delta	31.2922, 30.51599
	Ein Gedeirat	North Sinai	Sinai	30.65, 34.4333
	Ein Moussa	South Sinai	Sinai	29.8667, 32.65
	El-Arish	North Sinai	Sinai	31.1442, 33.8056
	El-Gebel El-Asfar	Qalyoubia	Lower Nile Valley and Delta	30.201416, 31.356162
	El-Katta	Giza	Lower Nile Valley and Delta	30.2191, 30.9678
	El-Magadlah	Giza	Lower Nile Valley and Delta	30.03986, 31.1046
	El-Mallah East	Cairo	Eastern Desert	30.8167, 32.1
	El-Wasfia	Ismailia	Eastern Desert	30.58076, 32.17235
	Esna	Qena	Upper Nile Valley	25.2919, 32.5532
	Ezbet Nakhl	Qalyoubia	Lower Nile Valley and Delta	31.1111, 32.1625
	Fayed	Ismailia	Eastern Desert	30.32382, 32.30079
	Fayoum	Fayoum	Fayoum	29.32061, 30.818
	Gebel Elba	Red Sea	Gebel Elba	22.2008, 36.3331
	Geneifa	Suez	Eastern Desert	30.1516, 32.429
	Gezira	Cairo	Lower Nile Valley and Delta	30.04596, 31.22435
	Girza	Fayoum	Fayoum	29.49968, 31.0738
	Giza	Giza	Lower Nile Valley and Delta	30.0135, 31.21127
	Halaib	Red Sea	Eastern Desert	22.2203, 36.6427
	Hawamdia	Giza	Lower Nile Valley and Delta	29.9092, 31.2615
	Helwan	Cairo	Lower Nile Valley and Delta	29.85, 31.3333
	Ismailia	Ismailia	Eastern Desert	30.59428, 32.26026
	Kafr Ghatati	Giza	Lower Nile Valley and Delta	30.00415, 31.12157
	Kafr Hakim	Giza	Lower Nile Valley and Delta	30.0808, 31.1164
	Kerdassa	Giza	Lower Nile Valley and Delta	30.0297, 31.1061
	Kom Ombo	Aswan	Upper Nile Valley	24.4761, 32.9483
	Kom Osheem	Fayoum	Fayoum	29.5564, 30.8869
	Kubba	Cairo	Lower Nile Valley and Delta	30.0876, 31.2854
	Maadi	Cairo	Lower Nile Valley and Delta	29.95772, 31.25054
	Madinet El-Sadat	Menofiya	Western Desert	30.35108, 30.51109
	Madinet Badr	Cairo	Lower Nile Valley and Delta	30.153, 31.7103
	Mansouriah	Giza	Lower Nile Valley and Delta	30.1236, 31.0725
	Mariout	Alexandria	Coastal Strip	31.0172, 29.76
	Mazghouna	Giza	Lower Nile Valley and Delta	29.7457, 31.2633
	Mersa Matrouh	Matrouh	Coastal Strip	29.5696, 26.4194
	Orman	Giza	Lower Nile Valley and Delta	30.02912, 31.21271
	Pyramids	Giza	Lower Nile Valley and Delta	29.9816, 31.1337
	Rafah	North Sinai	Sinai	31.2667, 34.2333
	Salloum	Matrouh	Coastal Strip	31.5888, 25.1026
	Saqqara	Giza	Lower Nile Valley and Delta	29.84955, 31.21641
	Sennouris	Fayoum	Fayoum	29.40662, 30.86602
	Sentris	Menofiya	Lower Nile Valley and Delta	30.31122, 31.0577
	Siwa Oasis	Matrouh	Western Desert	29.20427, 25.51928
	Suez	Suez	Eastern Desert	29.95278, 32.56582
	Tamiya	Fayoum	Fayoum	29.475479, 30.95228
	Turah	Cairo	Lower Nile Valley and Delta	29.9467, 31.2728
	W. Asal	Red Sea	Eastern Desert	25.9497, 34.383
	W. Dar El-Maskhara	Cairo	Eastern Desert	29.7833, 31.4167
	W. Digla	Cairo	Eastern Desert	29.9578, 31.3348
	W. El-Arbaein	South Sinai	Sinai	28.5469, 33.953
	W. El-Daiqa	North Sinai	Sinai	30.8278, 34.1401
	W. El-Lega	South Sinai	Sinai	28.5469, 33.953
	W. El-Natroun	Al-Behaira	Western Desert	30.3814, 30.3441
	W. El-Rahba	South Sinai	Sinai	28.82, 33.6419
	W. Firan	South Sinai	Sinai	28.7092, 33.3222
	W. Garawi	Cairo	Eastern Desert	29.7833, 31.3167
	W. Gedeirat	North Sinai	Sinai	30.6511, 34.4321
	W. Gharagid	Cairo	Eastern Desert	28.955, 31.4822
	W. Hoff	Cairo	Eastern Desert	29.8821, 31.311
	W. Ibib	Red Sea	Gebel Elba	22.83333, 35.76667
	W. Isla	South Sinai	Sinai	28.201, 34.244
	W. Morrah	Cairo	Eastern Desert	22.35, 33.75
	W. Rishrash	Giza	Eastern Desert	29.4642, 31.3672
	W. Um Elek	Cairo	Eastern Desert	29.8833, 31.5167
	W. Zohleiga	Cairo	Eastern Desert	26.1333, 33.75
Saudi Arabia	Abu-Arish	Jazan	Jazan	16.9821, 42.8389
	Al-Aqiq	Al-Aqiq	Al-Baha	20.2866, 41.6312
	Al-Mekhwa	Al-Mekhwa	Al-Baha	19.8429, 41.3115
	Ghabet Shahba	Al-Baha	Al-Baha	20.02723, 41.28565
	Jabal Shada al-A’la Nature Reserve	Al-Mekhwa	Al-Baha	19.8388, 41.3101
	Jazan	Jazan	Jazan	16.90245, 42.5705
	Jeddah	Jeddah	Makka Al-Mukarramah	21.5922, 39.2631
	Marabah	Marabah	Asir	18.0426, 42.3739
	Najran	Najran	Najran	17.4833, 44.1166
	Raydah Nature Reserve	Abha	Asir	18.20525, 42.41011
	Tabouk	Tabouk	Tabouk	28.36661, 36.629747
	Unayzah	Unayzah	Al-Qassim	26.08548, 43.9768
